# Approaching Dissolved Species in Ammonoacidic GaN Crystal Growth: A Combined Solution NMR and Computational Study

**DOI:** 10.1002/chem.201904657

**Published:** 2020-04-28

**Authors:** Peter Becker, Tanakorn Wonglakhon, Dirk Zahn, Dietrich Gudat, Rainer Niewa

**Affiliations:** ^1^ Institute of Inorganic Chemistry University of Stuttgart Pfaffenwaldring 55 70569 Stuttgart Germany; ^2^ Department of Chemistry and Pharmacy Friedrich Alexander University Erlangen-Nürnberg Nikolaus-Fiebiger-Str. 10 91058 Erlangen Germany

**Keywords:** ammonothermal synthesis, gallium, molecular dynamics simulations, nitrides, solution NMR

## Abstract

Solutions of gallium trihalides Ga*X*_3_ (*X*=F, Cl, Br, I) and their ammoniates in liquid ammonia were studied at ambient temperature under autogenous pressure by multinuclear (^71^Ga, ^35^Cl, ^81^Br) NMR spectroscopy. To unravel the role of pH, the analyses were done both in absence and in presence of ammonium halides, which are employed as mineralizers during ammonoacidic gallium nitride crystal growth. While gallium trifluoride and its ammoniate were found to be too sparingly soluble to give rise to a NMR signal, the spectra of solutions of the heavier halides reveal the presence of a single gallium‐containing species in all cases. DFT calculations and molecular dynamics simulations suggest the identification of this species as consisting of a [Ga(NH_3_)_6_]^3+^ cation and up to six surrounding halide anions, resulting in an overall trend towards negative complex charge. Quantitative ^71^Ga NMR studies on saturated solutions of GaCl_3_ containing various amounts of additional NH_4_Cl revealed a near linear increase of GaCl_3_ solubility with mineralizer concentration of about 0.023 mol GaCl_3_ per mol NH_4_Cl at room temperature. These findings reflect the importance of Coulombic shielding for the inhibition of oligomerization and precipitation processes and help to rationalize both the low solubility of gallium halides in neutral ammonia solution and, in turn, the proliferating effect of the mineralizer during ammonoacidic gallium nitride formation.

## Graphical abstract

**Materials synthesis**: A combination of solution NMR in liquid ammonia with quantum mechanical and molecular modelling allows identifying the dissolved species and their mineralizer concentration dependent solubility in ammonoacidic solutions with relevance for GaN crystal growth (see figure).

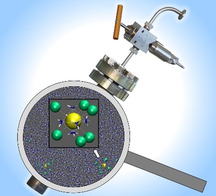

## Introduction

Ammonothermal synthesis is an emerging technique for preparation and crystal growth of many materials difficult to obtain. Pioneering work by *Robert Juza* and *Herbert Jacobs* starting in the 1960s already revealed the potential of the method for various material classes.^1^ In the past two decades, the technique gained great importance for growing high‐quality GaN crystals with particularly low defect rates,^2^ and it was recently further developed to produce various novel nitride‐based materials.^3^

For GaN crystal growth, two general routes have been established: The ammonobasic method typically employs alkali metal amides, or suitable precursors like alkali metals or alkali metal azides, as so‐called mineralizers to maintain a high concentration of amide (NH_2_^−^) ions, while the ammonoacidic method makes use of ammonium halides to boost the concentration of the ammonoacid NH_4_^+^.2b A successful ammonothermal synthesis depends crucially on the choice of these mineralizers to control the mobilization of starting and target materials as well as the solubility of any dissolved intermediates involved in the process. For a deeper understanding of the chemical processes, it is further essential to obtain concise knowledge on the species present in solution. Recently, we have been able to isolate several solid intermediates of ammonobasic GaN syntheses with different mineralizers, all of which contain isolated tetraamidogallate ions, [Ga(NH_2_)_4_]^−^.^4^ Furthermore, we have established the tetraamidogallate ion as the dominating dissolved species in liquid ammonia at ambient temperature and under autogenous pressure via solution NMR studies.^5^ Applying the heavier alkali metal amides as mineralizers may eventually afford ionic liquids with high gallium concentrations. Depending on the ammonia content, these liquids contain condensed μ‐imido amidogallate ions, which can be regarded as deprotonated intermediates towards crystalline GaN.^5^ In addition, molecular dynamics calculations have proven the complex ion [Ga(NH_2_)_4_]^−^ to represent the predominant species in liquid ammonia over a wide temperature and pH‐range.^6^

In ammonoacidic systems with ammonium halide mineralizers, we were similarly able to isolate solid intermediates of compositions [Ga(NH_3_)_6_]*X*_3_⋅NH_3_ (*X*=Br, I), [Ga(NH_3_)_5_Cl]Cl_2_, and [Ga(NH_3_)_4_F_2_][Ga(NH_3_)_2_F_4_]=Ga(NH_3_)_3_F_3_, respectively.^7^ The heavier halide anions (bromide and iodide) give rise to salts containing hexaammine gallium ions, while the lighter halides (chloride and fluoride) are able to enter the first coordination sphere of gallium. Remarkably, the crystalline ammoniate Ga(NH_3_)_3_F_3_ features both isolated cationic and anionic complexes. Molecular dynamics calculations suggest that such ions may persist in liquid ammonia at ambient pressure, although with an increased number of ammonia ligands in the first coordination sphere of gallium, and agglomerate upon heating and pressurizing, due to the diminishing permittivity of ammonia under these conditions.^8^ Unfortunately, the extremely low solubility of this complex fluoride in ammonia at ambient temperature thwarted as yet any experimental studies.

Herein, we report on NMR studies of ammonacidic solutions of gallium salts in ammonia at ambient temperature and under autogenous pressure, which aim at pinpointing the solute species present. The results are interpreted based on DFT‐calculations of the spectroscopic data and discussed in the context of molecular dynamics simulations in a wide temperature and pressure range.

## Results and Discussion

For our NMR experiments, gallium halides Ga(NH_3_)_3_F_3_ (**1**), [Ga(NH_3_)_6_]Br_3_⋅NH_3_ (**2**) and [Ga(NH_3_)_6_]I_3_⋅NH_3_ (**3**) were prepared as described in the experimental section and used, after characterization as single phases by powder X‐ray diffraction, for the preparation of solutions in liquid ammonia.7a Production of single phase [Ga(NH_3_)_5_Cl]Cl_2_ via ammonoacidic synthesis was unfortunately not successful. Instead, we investigated solutions of GaCl_3_ (**4**) in liquid NH_3_, which are expected to deliver the same dissolved species.

All gallium trihalides are known to exhibit quite low solubilities in liquid ammonia around room temperature.^9^ Similarly, we found that ammine complexes [Ga(NH_3_)_6_]Br_3_ **⋅**NH_3_ (**2**) and [Ga(NH_3_)_6_]I_3_ **⋅**NH_3_ (**3**) are only sparingly soluble in pure liquid ammonia, and most of the samples prepared contained a solid residue next to the saturated solution. Nonetheless, ^71^Ga NMR signals of these solutions were readily observable, and recording of a ^71^Ga NMR spectrum of the ammonia solution of GaCl_3_ (**4**) was likewise feasible after prolonged acquisition. In case of Ga(NH_3_)_3_F_3_ (**1**), neither ^71^Ga nor ^19^F NMR signals were detectable. Interpretation of this last finding requires some concern about the effect of spin coupling on the spectra. Reported values of ^1^ *J*_71Ga,19F_ range from 264 Hz in (NH_4_)_3_[GaF_6_] to 445–490 Hz in [(NNN)GaF_3_] (NNN=triazacyclononane ligand),^10^ and observable splitting of signals into multiplets is thus to be expected when intermolecular fluoride exchange is slow and a small electric field gradient at the metal center precludes rapid relaxation induced by the quadrupolar nature of the two naturally occurring gallium isotopes ^69, 71^Ga (both *I*=3/2). NMR studies on fluoro‐gallium complexes are scarce, but indicate that ^19^F NMR signals seem readily detectable even if linewidths may in unfavorable cases exceed 1 kHz.^11^ Against this background, we conclude that the failure to detect any ^19^F NMR signals from **1** is most likely an indication that the complex is essentially insoluble.

The ^1^H and ^14^N NMR spectra of solutions of **2**–**4** (as well as all other samples included in this study) display a single resonance representing a dynamic average of the signals of bound and free NH_3_ molecules. Exchange of this type is known for aluminum complexes, and the lifetimes observed for bound ligands in that case (≈1 s at −37 °C)^12^ are in accord with the assumption that the reaction has reached the fast exchange regime at ambient temperature.

**Figure 1:**
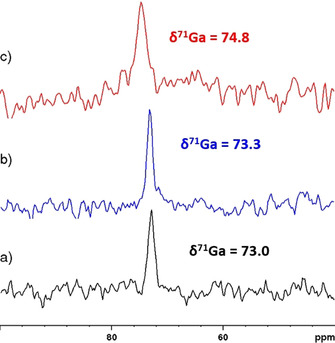
^71^Ga NMR spectra of saturated solutions of (a) GaCl_3_ (**4**), (b) [Ga(NH_3_)_6_]Br_3_⋅NH_3_ (**2**) and (c) [Ga(NH_3_)_6_]I_3_⋅NH_3_ (**3**) in liq. NH_3_ at room temperature. Chemical shifts and linewidths (Δ*ν*_1/2_=114 Hz (**4**), 85 Hz (**2**), 213 Hz (**3**)) were obtained from spectral deconvolution.

The ^71^Ga NMR spectra of solutions of **2**–**4** display single lines (Figure 1) with very similar chemical shifts around 74 ppm and moderate line widths, indicating that all three solutions contain a common, highly symmetrical gallium‐containing species. Based on previous reports on the presence of six‐coordinate complexes in solutions of aluminum(III) halides in liq. NH_3_ 13 and isolated gallium halide ammoniates7a, 9b as well as aqueous solutions of gallium(III) salts,7a, 14 we tentatively appoint this species as a hexa‐coordinated complex [Ga(NH_3_)_6_]^3+^. A further discussion of this assignment in the light of computational studies will be given further below.

**Figure 2:**
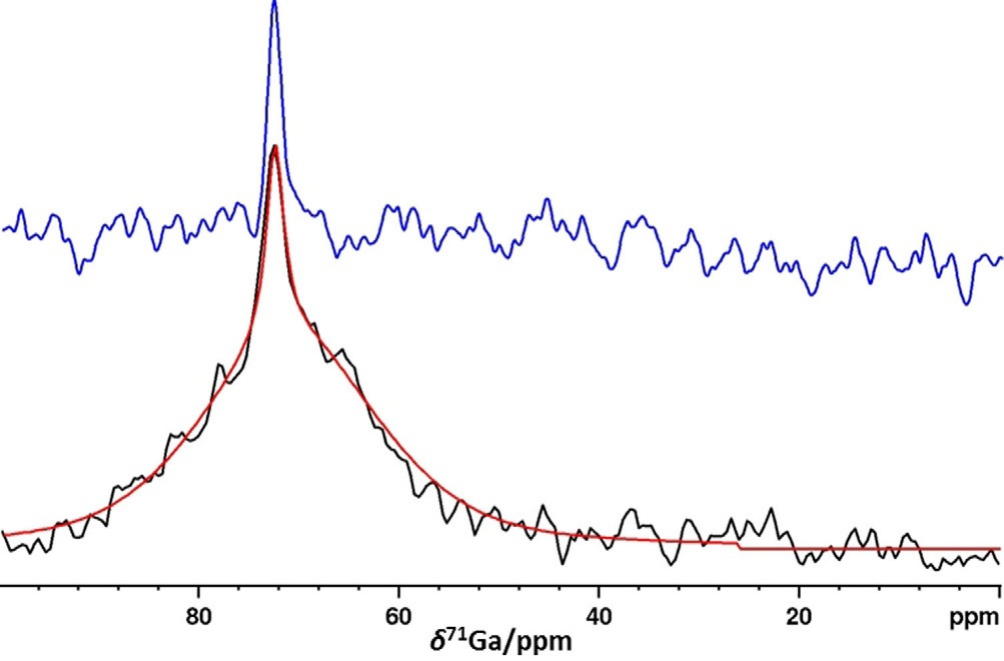
Room temperature ^71^Ga NMR spectrum of a solution of GaCl_3_ (**4**) in liq. NH_3_ with a sediment of undissolved salt (black trace) and result of a fit (red trace) as two superimposed lines at δ^71^Ga=72.7 ppm (Δ*ν*_1/2_=262 Hz, 10 %) and 71.3 ppm (Δ*ν*_1/2_=2590 Hz, 90 %). The blue trace represents the spectrum of a filtered solution without sediment. Both spectra were processed by applying an exponential apodization function with a line broadening factor of 100 Hz prior to FT.

The ^71^Ga NMR spectrum of a solution of **4** that contains a sediment of undissolved material features, in addition to the signal already observed for the clear solution, a second resonance with a similar chemical shift but a significantly larger line width (Figure 2). We presume that this signal arises from a species present at the interface between the solid and the supernatant solution. In view of the relation between the linewidths in NMR spectra of quadrupolar nuclei like ^71^Ga (*I*=3/2, *Q*=0.106 barn) and both local electric field gradients and rotational correlation times,^15^ the increased linewidth of the additional resonance can in principle be explained as arising from the reduced mobility of a surface species, or the presence of a less symmetrical coordination sphere than in a [Ga(NH_3_)_6_]^3+^ complex in the bulk solution, respectively.

A likely origin of a lower local symmetry is the incorporation of chloride ligands in the metal coordination sphere. A first attempt to confirm this hypothesis by characterizing the chemical environment of the chlorine atoms by ^35^Cl NMR spectroscopy failed, however, as low solute concentration and/or coordination‐induced line broadening precluded the observation of any signals above noise level. Assuming that the formation of heteroleptic complex ions like [Ga(NH_3_)_5_Cl]^2+^ as found in solid [Ga(NH_3_)_5_Cl]Cl_2_ can be promoted by the addition of excess ligand, we extended our studies to solutions of mixtures of GaCl_3_ and an ammonium halide (NH_4_Cl) in liquid ammonia. Moreover, since addition of NH_4_Cl lowers the pH value, this approach offers an opportunity to study the behavior of the gallium salt not only in neutral, but also in ammonoacidic milieu. Considering that salt addition affects also the ion strength of the solutions, we chose to use both NH_4_Cl and NH_4_Br as mineralizer (the absence of detectable amounts of bromo‐complexes in solutions of **2** led us to consider bromide as “innocent” with respect to complex formation) in order to separate both effects.

**Figure 3:**
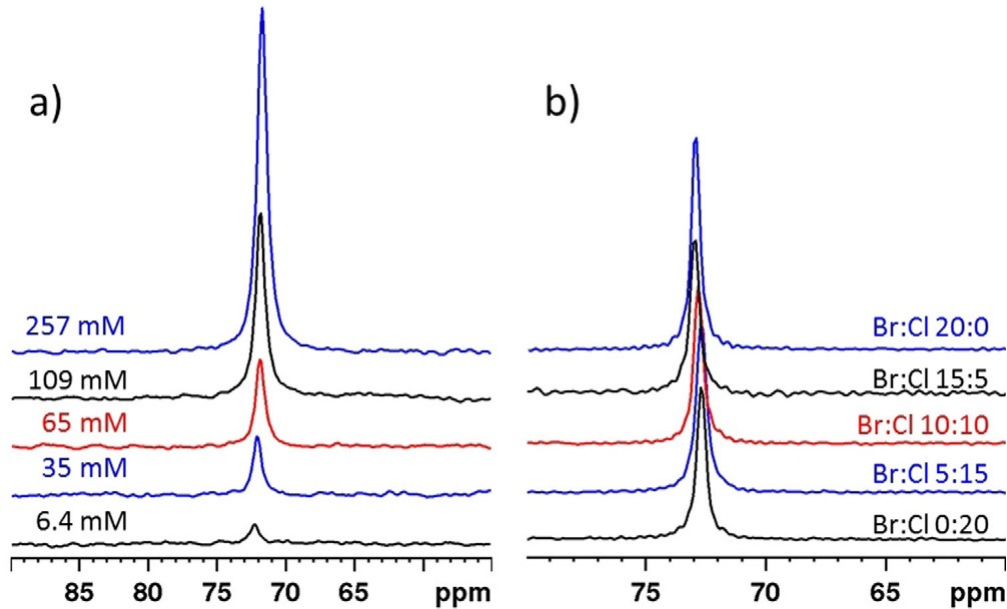
Room temperature ^71^Ga NMR spectra (a) of saturated solutions of GaCl_3_ in liq. NH_3_ containing varying concentrations of NH_4_Cl, (b) of GaCl_3_ solutions in liq. NH_3_ containing different molar ratios of NH_4_Br and NH_4_Cl. All spectra displayed in (a) were recorded and processed using identical protocols and are drawn to scale.

Somewhat unexpected, the ^71^Ga NMR spectra of saturated solutions of GaCl_3_ in liquid ammonia containing varying quantities of NH_4_Cl gave no evidence for the formation of any new species, but revealed merely a rise in signal intensity with increasing ammonium halide concentration (Figure 3 a; note that the spectra of samples prepared by incomplete dissolution of **4** in the presence of NH_4_Cl displayed a similar additional broad resonance as samples obtained without mineralizer, see Supporting Information). Variation of the nature of the anion in solutions containing similar total concentrations of NH_4_ *X* (*X*=Cl, Br) had no impact on chemical shifts or linewidths (Figure 3 b), suggesting that the halide ions do not enter the first metal coordination sphere. This conjecture is further corroborated by the observation of narrow lines indicating the presence of non‐coordinated halide anions in the ^35^Cl and ^81^Br NMR spectra of these solutions.

**Figure 4:**
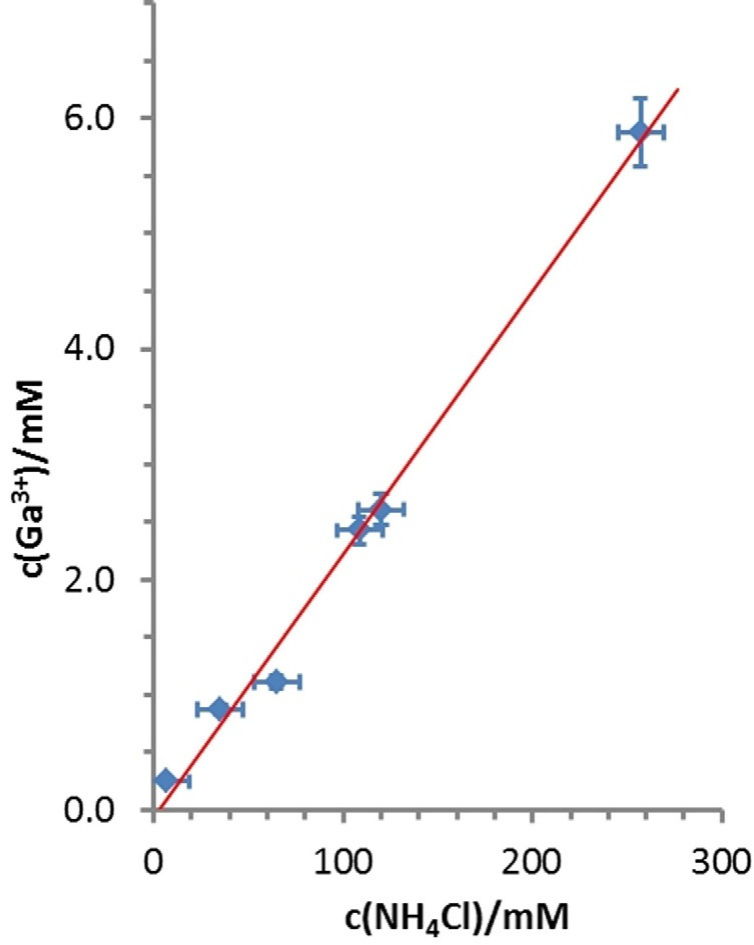
Plot of gallium concentration (determined from quantitative ^71^Ga NMR measurements) vs. mineralizer concentration for saturated solutions of GaCl_3_ in liq. NH_3_ at room temperature. Further methodical details are given in the experimental section.

A plot of gallium concentrations determined from quantitative ^71^Ga NMR measurements on saturated solutions versus the concentration of NH_4_Cl added (Figure 4) suggests a linear correlation between both quantities which implies a similar dependence of gallium solubility on mineralizer concentration as had previously been established for GaN in supercritical ammonia at 490 °C.^16^ The molar amount of GaCl_3_ dissolved per mole of mineralizer can be computed from the slope of a linear regression curve as 0.023(14) mol GaCl_3_ per mol NH_4_Cl. This result comes close to a reported value of 0.05 mol GaCl_3_ per mol NH_4_Cl for the solubility of GaN in NH_4_Cl‐containing supercritical ammonia at 550 °C determined by in situ X‐ray imaging,^8^, ^17^ but is far from figures of up to 0.43 mol‐% GaN per mol‐% NH_4_Cl at temperatures between 400 and 600 °C that had been obtained by a gravimetric method.^16^ While any quantitative comparison of the data from the diverse sources seems problematic in view of the differences in methods as well as concentration and temperature regimes involved, our results nonetheless further corroborate the previous findings on the importance of mineralizer concentration as a decisive factor for the solubility of gallium salts in ammonoacidic milieu.^8^, ^16^, ^17^ For closer comparison of the various figures, it is noteworthy that the solubility of GaN in presence of ammonium halides will strongly depend on the properties of ammonia, which are known to significantly change with temperature and pressure, particularly in vicinity of the critical point. Next to a significant decomposition of ammonia to form hydrogen and nitrogen,^18^ thus reducing the solvent concentration, the decreasing permittivity of ammonia with increasing temperature has to be taken into account.

To substantiate the nature of the complexes present in solution and assess the effects of chloride coordination and protolysis on ^71^Ga NMR chemical shifts, we performed DFT calculations on a series of tentative structural candidates, namely [Ga(NH_3_)_*n*_]^3+^ (*n=*4–6), [GaCl_*n*_]^(*n*−3)−^ (*n=*5, 6), [Ga(NH_3_)_6−*n*_(NH_2_)_*n*_]^(3−*n*)+^ (*n=*1–3), and [Ga(NH_3_)_*n*_ *X*_6−*n*_]^(3−*n*)−^ (*X*=Cl, Br, I; *n=*1–3), [Ga(NH_3_)_*n*_ *X*_6−*n*_]^(*n*−3)+^ (*X*=Cl; *n=*4, 5), respectively. Considering that the low dielectric permittivity of liquid ammonia might favor ion pairing, we further evaluated δ^71^Ga for ion assemblies {[Ga(NH_3_)_6_]Cl_*n*_}^(3−*n*)+^ (*n=*1–3), {[Ga(NH_3_)_6_]Cl_*n*_}^(*n*−3)−^ (*n=*4–6) and {[Ga(NH_3_)_5_(NH_2_)]Cl_2_}, respectively. Complexes with higher metal coordination numbers were neglected, since attempts to locate energy optimized geometries for such species converged inevitably to the structures of van der Waals complexes between hexa‐coordinate complexes and additional, loosely interacting, ammonia molecules.

**Figure 5:**
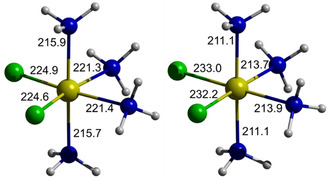
Ball‐and‐stick representations of the energy optimized ωB97X‐D/def2‐tzvp local minimum structures of *cis*‐[Ga(NH_3_)_4_Cl_2_]^+^ in the gas phase (left) and in liquid ammonia (simulation of solvation effects by a PCM model, right). The figures represent bond distances in pm. Colors: Ga (yellow), Cl (green), NH_3_ (blue/white).

The molecular structures used for these studies were obtained by performing first energy optimizations on isolated complexes or ion assemblies (in the „gas phase′), and then re‐optimizing the initial structures under application of the PCM (polarizable continuum model)^19^ approach for modelling the effect of solvation. Comparison of „gas phase“ and „solute“ structures (see Figure 5 for an example) discloses that embedding in the dielectric medium tends to lengthen Ga–Cl and shorten Ga–N distances, which implies a significant electrostatic contribution to the metal‐ligand bonding. The chloride anions in ion assemblies {[Ga(NH_3_)_6_]Cl_*n*_}^(3−*n*)+^ (*n=*1–3) and {[Ga(NH_3_)_6_]Cl_*n*_}^(*n*−3)−^ (*n=*4–6) display each three close contacts to NH‐bonds of coordinated ammine ligands (Cl⋅⋅⋅H 222–249 pm, N⋅⋅⋅Cl 322–336 pm) with an essentially linear arrangement of the N−H⋅⋅⋅Cl moieties. The distances match those reported for NH⋅⋅⋅Cl hydrogen bonds in organic crystals (320–334 pm for NH⋅⋅⋅Cl and NH^+^⋅⋅⋅Cl units based on the sum of hydrogen bond radii^20^ and the same structural motif with N⋅⋅⋅Cl distances ranging from 326.7(2)–352.4(3) pm was also found in crystal structures of some hexammine cobalt complexes.^21^ Attachment of chlorides induces structural distortions, which are evidenced by a larger variance of individual Ga–N distances in some assemblies and a general contraction of the coordination polyhedra with increasing number of anions (cf. Figure S4 and the extreme values of 213.2 pm and 209.5 pm for average Ga–N distances in [Ga(NH_3_)_6_]^3+^ and {[Ga(NH_3_)_6_]Cl_6_}^3−^).

Subsequent magnetic shielding calculations were carried out using the zero order regular approximation (ZORA)^22^ in order to account in a consistent manner for spin‐orbit induced effects associated with the presence of heavy atom substituents^23^ (*X*=Br, I). Further details on the computations are given in the experimental section, and a listing of calculated ^71^Ga chemical shifts and the square of the largest component of the electric field gradient (efg) tensor, *q*_zz_^2^, which has been shown to correlate in favorable cases with observed spectral linewidths,^24^ is presented in Table 1.

**Table 1:** Calculated values of δ^71^Ga_calcd_ (in ppm) and *q*_zz_^2^ (in a.u.) for some gallium complexes containing ammine and halide ligands (see experimental section for technical details).

Species	δ^71^Ga_calcd_	*q*_zz_^2^
[Ga(NH_3_)_4_]^3+^	302.3	4.0×10^−14^
[Ga(NH_3_)_5_]^3+^	182.7	1.3×10^−2^
[Ga(NH_3_)_6_]^3+^	62.5	8.4×10^−4^
		
{[Ga(NH_3_)_6_]Cl}^2+^	69.6	5.3×10^−4^
{[Ga(NH_3_)_6_]Br}^2+^	68.2	5.3×10^−4^
{[Ga(NH_3_)_6_]I}^2+^	70.0	9.6×10^−4^
		
{[Ga(NH_3_)_6_]Cl_2_}^+^	71.8	4.0×10^−3^
{[Ga(NH_3_)_6_]Br_2_}^+^	70.6	4.0×10^−3^
{[Ga(NH_3_)_6_]I_2_}^+^	73.8	4.0×10^−3^
		
{[Ga(NH_3_)_6_]Cl_3_}	74.5	4.5×10^−3^
{[Ga(NH_3_)_6_]Br_3_}	72.6	3.6×10^−3^
{[Ga(NH_3_)_6_]I_3_}	77.4	3.4×10^−3^
		
{[Ga(NH_3_)_6_]Cl_4_}^−^	80.5	3.1×10^−3^
{[Ga(NH_3_)_6_]Cl_5_}^2−^	78.1	2.4×10^−2^
{[Ga(NH_3_)_6_]Cl_6_}^3−^	80.1	3.0×10^−2^
		
[Ga(NH_3_)_5_(NH_2_)]^2+^	80.1	1.2
{[Ga(NH_3_)_5_(NH_2_)]Cl_2_}	84.5	0.33
		
*cis*‐[Ga(NH_3_)_4_(NH_2_)_2_]^+^	112.4	1.1
*trans*‐[Ga(NH_3_)_4_(NH_2_)_2_]^+^	100.7	4.8
		
[Ga(NH_3_)_5_Cl]^2+^	68.1	5.3×10^−3^
[Ga(NH_3_)_5_Br]^2+^	20.4	9.6×10^−4^
[Ga(NH_3_)_5_I]^2+^	−66.5	6.1×10^−3^
		
*cis*‐[Ga(NH_3_)_4_Cl_2_]^+^	62.1	4.0×10^−3^
*trans*‐[Ga(NH_3_)_4_Cl_2_]^+^	73.3	1.6×10^−5^
*cis*‐[Ga(NH_3_)_4_Br_2_]^+^	−53.9	1.5×10^−2^
*trans*‐[Ga(NH_3_)_4_Br_2_]^+^	−51.7	1.8×10^−2^
*cis*‐[Ga(NH_3_)_4_I_2_]^+^	−301.8	1.8×10^−2^
*trans*‐[Ga(NH_3_)_4_l_2_]^+^	−164.9	9.9×10^−2^
		
mer‐[Ga(NH_3_)_3_Cl_3_]	87.0	4.0×10^−2^
fac‐[Ga(NH_3_)_3_Cl_3_]	70.2	7.3×10^−4^
mer‐[Ga(NH_3_)_3_Br_3_]	−109.4	0.11
fac‐[Ga(NH_3_)_3_Br_3_]	−173.6	3.2×10^−4^
mer‐[Ga(NH_3_)_3_I_3_]	−480.5	0.19
fac‐[Ga(NH_3_)_3_l_3_]	−663.5	7.4×10^−3^
		
*cis*‐[Ga(NH_3_)_2_Cl_4_]^−^	23.5	8.4×10^−2^
*trans*‐[Ga(NH_3_)_2_Cl_4_]^−^	26.8	0.18
		
[Ga(NH_3_)Cl_5_]^2−^	−5.2	0.17
		
[GaCl_5_]^2−^	99.4	4.6×10^−2^
[GaCl_6_]^3−^	−69.2	0.0

The trend in calculated ^71^Ga chemical shifts of ammine complexes [Ga(NH_3_)_*n*_]^3+^ (*n=*4–6) reflects the expected^25^ strong influence of the coordination number on the magnetic shielding of the metal atom, but the dependence of δ^71^Ga_calcd_ on the number and nature of the halide anions in the aggregates {[Ga(NH_3_)_6_]*X_n_*} emphasizes that changes in the second coordination sphere have as well a visible impact. Without intending a quantitative assessment (which would presumably require averaging over a large number of structural isomers with different arrangement of the surrounding anions^26^), we note that δ^71^Ga_calcd_ of the ion clusters increases generally—although not linearly—with the number of surrounding halide anions, and that the magnitude of the deshielding effect grows in the order Br<Cl<I. These trends are presumably related to the structural distortions of the cationic core arising from the H‐bonding and electrostatic interactions with the surrounding anions.

Detachment of one or two protons to produce the amido‐complexes [Ga(NH_3_)_6−*n*_(NH_2_)_*n*_]^(3−*n*)+^ (*n=*1, 2), induces moderate or significant increases in δ^71^Ga_calcd_, respectively. Simulation of a dimeric complex [(NH_3_)_4_Ga(μ‐NH_2_)_2_Ga(NH_3_)_4_]^4+^ featuring two edge‐sharing GaN_6_ octahedra revealed that the condensation had, as in the case of amidogallates,^5^ nearly no effect on the chemical shift (δ^71^Ga_calcd_=78.8 ppm compared to 80.1 ppm for [Ga(NH_3_)_5_(NH_2_)]^2+^). Attempting to establish as well a molecular geometry for a hypothetical neutral species [Ga(NH_3_)_3_(NH_2_)_3_], we found that energy optimization runs converged inevitably to structures that are best described as ammoniates of gallium amide, [Ga(NH_2_)_3_(NH_3_)], with tetrahedrally coordinated Ga. The emergence of such species, which formally represent the conjugate acid of the previously identified^5^ amidogallate [Ga(NH_2_)_4_]^−^, in the computational studies indicates that the hexa‐coordinate monomeric complexes may become eventually unstable at elevated pH.

**Figure 6:**
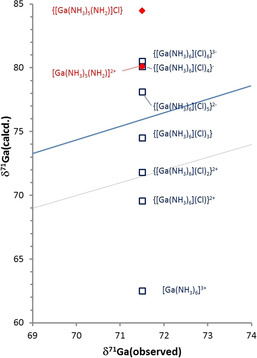
Correlation between the observed ^71^Ga NMR chemical shift of GaCl_3_/NH_4_Cl solutions in liquid ammonia and calculated chemical shifts of model systems {[Ga(NH_3_)_6_]Cl_*n*_}^(3−*n*)+^ (*n=*1–3) and {[Ga(NH_3_)_6_]Cl_*n*_}^(*n*−3)−^ (*n=*4–6; blue squares) and {[Ga(NH_3_)_5_(NH_2_)]Cl_*n*_}^(2−*n*)+^ (*n=*0, 2; red diamonds). The solid blue line denotes the result of a linear correlation between observed and calculated chemical shifts of reference compounds,^5^ and the dashed grey line the relation δ^71^Ga_obs_=δ^71^Ga_calcd_.

The computed ^71^Ga chemical shifts for chlorogallium complexes [Ga(NH_3_)_*n*_Cl_6−*n*_]^(*n*−3)+^ (*n=*3–5) are quite similar or—in case of *trans*‐[Ga(NH_3_)_4_Cl_2_]^+^ and fac/mer‐[Ga(NH_3_)_3_Cl_3_]—slightly larger than that of the “naked” hexaammine complex, whereas the chemical shifts of the homologous bromo‐ and iodo‐complexes are substantially lower and tend to become increasingly more negative with growing atomic size (thus showing a normal halogen dependence and number of halide ligands).^27^ An inspection of individual shielding contributions reveals that the differential changes in δ^71^Ga_calcd_ for complexes containing exclusively amine‐/amide‐ and chloride‐based ligands depend mainly on variations in the paramagnetic shielding term,^28^ whereas the trends in complexes with metal‐bound bromide and iodide ions are dominated by spin‐orbit effects.^23^ Quite surprising, incorporation of halide ligands into the first coordination sphere induces in most cases no sharp increase in the magnitude of *q*_zz_^2^, which implies that the effective local electron density distribution around the metal ion remains quite symmetrical. In view of this finding, it is tempting to interpret the discrepancy between computed „gas phase“ and „solute“ geometries (Figure 5) as the consequence of a structural adaptation to the polarizable continuum that is driven by the demand to establish a maximum symmetrical charge distribution. The spread of computed chemical shifts for all complexes considered in Table 1 corroborates our initial hypothesis of interpreting the coincident values of δ^71^Ga for solutions of **2**–**4** in liquid ammonia (Figure 1) as an indication for the presence of the same homoleptic complex in all cases. A numerical comparison suggests further that structures with tetra‐ or penta‐coordinate gallium ions can definitely be ruled out and the experimental data are best compatible with a hexa‐coordinate species. Even so, the assignment of the solute species to a “naked” [Ga(NH_3_)_6_]^3+^ ion implies an unusually large deviation from an empirical regression curve connecting observed and calculated chemical shifts (Figure 6 and ESI). In principle, two straightforward explanations of this discrepancy can be conceived, viz. the sensitivity of δ^71^Ga_calcd_ to changes in the second coordination sphere, or acid‐base reactions implying deprotonation of one (or more) of the NH_3_ ligands, respectively. Considering the presence of ionic aggregates {[Ga(NH_3_)_6_]*X_n_*}^(3−*n*)+^ (*n=*1–3; *X*=Cl, Br, I) and {[Ga(NH_3_)_6_]Cl_*n*_}^(*n*−3)−^ (*n=*4–6, see also Figure 6 for *X*=Cl) is indeed suited to improve the conformity with the correlation, with the best match with a single species achievable in case of **4** for ion clusters of compositions {[Ga(NH_3_)_6_]Cl_3_} and {[Ga(NH_3_)_6_]Cl_5_}^2−^, respectively. A similar improvement is feasible by assuming that the solutions contain rapidly exchanging dynamic mixtures consisting of substantial amounts of both the hexaammine complex and its conjugate base, [Ga(NH_3_)_5_(NH_2_)]^2+^. However, this hypothesis is in conflict with the expectation that the addition of varying amounts of NH_4_ *X* should then induce changes in the equilibrium composition and thus the observable average chemical shift, which is actually not observed. We conclude therefore that postulating the presence of ion clusters provides the most likely scenario for solutions containing additional ammonium halide as mineralizer, although it cannot be excluded that formation of amido‐complexes may compete with ion pairing in solutions of gallium halides in pure ammonia. The modulation of the local environment of the metal center by dynamic exchange of anions in the second coordination sphere may well be considered to contribute to the observed variation in ^71^Ga linewidths. It must be admitted, however, that the limited accuracy of the computational model precludes a reliable evaluation of the number of attached anions and that, in view of the rather minute impact of the variation of the nature and number of halide anions surrounding a complex cation on the resulting chemical shift, we cannot exclude that the real solution may not contain a single species but rather a dynamically equilibrating mixture in which variations in δ^71^Ga are levelled beyond the limit of experimental observability.

While the DFT calculations provide a very accurate account of the interactions within model complexes, the consideration of the embedding solvent lacks explicit atomic detail. To permit unbiased exchange of ammonia molecules between solvent shells and the bulk solution, we hence employed molecular mechanics based molecular dynamics (MD) simulations. On this basis, bulk ammonia solutions are mimicked by periodic simulation cells of 3000 NH_3_ molecules at 300 K and 8 atm. Following a recently introduced ‘local p*K*’ modelling scheme,^6^ we use combined quantum/molecular mechanics to account for ammonium/amide acid/base reactions as functions of complex formation and pH. On this basis, we recently calculated the local p*K* of the [Ga(NH_2_)_4_]^−^/ [Ga(NH_2_)_3_(NH_3_)]^0^ reaction, and clearly established [Ga(NH_2_)_4_]^−^ as the preferred complex by 44 kcal mol^−1^. This implied shifting the p*K* of ammonia from 32 to 1 when contrasting autoprotolysis in bulk ammonia to Ga^3+^ assisted NH_3_ protolysis.^6^

To elucidate the role of Cl^−^ ions, it is educative to directly compare [Ga(NH_2_)_4_]^−^ with the analogous [GaCl_4_]^−^ complex in ammonia solution. Strikingly, we find the chloride complex to quickly evolve from nearest‐neighbor Ga–Cl contacts towards {[Ga(NH_3_)_6_]Cl_4_}^−^ species. The latter type of complexes predominantly exhibit Cl^−^ ions in the second coordination shell of Ga (with an average Ga–Cl distance of 350 pm) whereas nearest‐neighbor Ga–Cl contacts (Ga–Cl distance of 280–300 pm) are only observed as temporary fluctuations. The stability of the hexaammine motif, [Ga(NH_3_)_6_]^3+^ drastically depends on the presence of Cl^−^ in the second coordination shell. Contrasting the two speculative systems “[Ga(NH_2_)_4_]^−^+*n* Cl^−^” and “{[Ga(NH_3_)_6_]Cl_*n*_}^(*n*−3)−^+4 (NH_2_)^−^” (with *n* ≥3), we find the ammine complex to be favored by more than 100 kcal mol^−1^, hence fully overcompensating the beforehand discussed promotion of amide formation next to dispersed Ga^3+^ ions.

**Figure 7:**
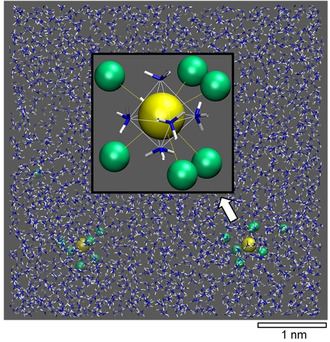
Snapshot from a molecular dynamics run of two {[Ga(NH_3_)_6_]Cl_6_}^3−^ complexes forming a stable dispersion in ammonia solution. The chloride ions are arranged in the second coordination shell of the gallium ions above the faces of the [Ga(NH_3_)_6_]^3+^ octahedra and engage, in addition to the Coulombic interaction with the Ga^3+^ ion, in hydrogen bonding with ammonia molecules in both the metal complex and the embedding solvent (H⋅⋅⋅Cl 240–250 pm). While full dissociation from the complex is not observed within the ns scale of the molecular dynamics runs, residence times above specific triangular motifs are only around 3 ps. Both, association of further chloride ions and complex dimerization is disfavored by repulsive Coulomb interactions. Colors: Ga (yellow), Cl (green), NH_3_ (blue/white).

We then placed two {[Ga(NH_3_)_6_]Cl_*n*_}^(*n*−3)−^ type complexes in our models of ammonia solution (without charge compensation) to explore trends towards agglomeration. Indeed, in our small simulation cell of approx. 5×5×5 nm^3^ dimensions, we find the association of {[Ga(NH_3_)_6_]Cl_*n*_}{[Ga(NH_3_)_6_]Cl_*m*_}^(6−*n‐m*)+^ dimer complexes for *n*, *m*<4 within a few 100 ps. On the other hand, for *n*≥4 the {[Ga(NH_3_)_6_]Cl_*n*_}^(*n*−3)−^ type species displayed stable dispersions during the entire length of the MD runs (5 ns). To account for the range of ammonium chloride concentrations added to the GaCl_3_ solution, we probed the fate of {[Ga(NH_3_)_6_]Cl_*n*_}^(*n*−3)−^ complexes up to *n=*7. From this, *n=*6, that is the {[Ga(NH_3_)_6_]Cl_6_}^3−^ complex (Figure 7), was identified as the most negatively charged species to which no further Cl^−^ could be associated. Based on the MD simulations, we hence argue that {[Ga(NH_3_)_6_]Cl_3_}^0^ complexes tend to form oligomers because of energetic favoring. To get stable dispersion, entropic favoring stemming from strong dilution (much more than the modelled ratio of 1500 NH_3_ per Ga) is required. This explains the comparably low solubility of GaCl_3_ in the absence of NH_4_Cl. In turn, already an equimolar solution of GaCl_3_ and NH_4_Cl leads to {[Ga(NH_3_)_6_]Cl_4_}^−^ complexes that show Coulombic shielding against dimerization. Such stabilization may be further boosted by increasing the NH_4_Cl content—with the maximum being represented by the dispersion of {[Ga(NH_3_)_6_]Cl_6_}^3−^ complexes.

## Conclusions

Our studies on solutions of gallium halides Ga*X*_3_ (*X*=Cl, Br, I) and their ammoniates in liquid ammonia at ambient temperature and under autogenous pressure reveal the presence of a small concentration of a single common gallium‐containing species. The concentration of this species increases upon addition of ammonium halides as mineralizers. Even if the process conditions do not align with GaN crystal growth and neglect important factors like change of properties and decomposition of ammonia, quantitative ^71^Ga NMR studies imply that the gallium saturation concentration displays a similar linear increase with mineralizer concentration as had previously been established for the solubility of GaN in supercritical ammonia at high temperatures.^8^, ^16^, ^17^ The observation of a further broad ^71^Ga NMR signal in solutions containing a solid deposit of GaCl_3_ is tentatively assigned to a surface species which could not be unequivocally identified.

Comparison of the experimental data with computationally predicted chemical shifts (from DFT calculations) of homoleptic ([Ga(NH_3_)_*n*_]^3+^ with *n*=4–6) and heteroleptic ([Ga(NH_3_)_*n*_ *X*_6−*n*_]^(3−*n*)−^ with *X*=Cl, Br, I; *n*=1–3 and [Ga(NH_3_)_*n*_ *X*_6−*n*_]^(*n*−3)+^ with *X*=Cl; *n*=4–5) complexes suggests to identify the dissolved species as ion clusters {[Ga(NH_3_)_6_]*X_n_*} with an unspecified number of halide anions. Moreover, molecular dynamics simulations dedicated to bulk ammonia solutions of the GaCl_3_–NH_4_Cl–NH_3_ system predict that anionic clusters, with {[Ga(NH_3_)_6_]Cl_6_}^3−^ as the most negatively charged species, are energetically preferred and display stable dispersion in solution, while neutral complexes {[Ga(NH_3_)_6_]Cl_3_} tend to aggregate.

Considering all our findings, we conclude that neutral or ammonoacidic solutions of gallium halides in liquid ammonia contain complexes {[Ga(NH_3_)_6_]*X_n_*}^(*n*−3)−^ (with *n=*4–6 in the presence of NH_4_ *X* as mineralizers) as majority species. Apart from indicating the importance of Coulombic shielding for the inhibition of oligomerization and precipitation processes, these results allow us to rationalize both the low solubility of gallium halides in neutral ammonia and the proliferating effect of the mineralizer. The DFT calculations indicate further that incorporation of halide ions in the first coordination sphere around gallium strongly affects chemical shifts, with trends in δ^71^Ga_calcd_ depending mainly on variations in the paramagnetic shielding term for complexes containing only nitrogen‐ and chloride‐based ligands and on spin‐orbit effects for complexes with bromide and iodide ligands, whereas the effective local electron density distribution around the metal ion (as expressed by *q*_zz_^2^) remains quite symmetrical. We explain this, at first glance surprising, finding as a response of the complexes to the polarizing effect of the dielectric medium in terms of structural relaxation of metal–ligand distances in the first coordination sphere.

While these results all hold for solutions of gallium halides in ammonia with or without presence of excess ammonia halides at ambient temperatures, we have to hold in mind changes of solvent properties, particularly decreasing permittivity with increasing temperature and decomposition of ammonia, reducing the available concentration of solvent, if conclusions for ammonothermal GaN crystal growth are derived. Still, we strongly believe that these investigations open the door to a deeper understanding of the chemical processes involved. It, however, remains a major challenge to carry the NMR investigations to supercritical fluids for a closer approach to the chemical conditions relevant for GaN crystal growth.

## Experimental Section

### Synthesis

The gallium halides were purchased from Sigma Aldrich (purity ≥99,999 % trace metals basis, anhydrous). All manipulations were carried out under argon (glove‐box: MBraun, Garching, Germany, *p*(O_2_)<0.1 ppm). The ammoniates were synthesized as described by Zhang et al.7a [Ga(NH_3_)_6_]*X*_3_ with *X*=Br, I were synthesized by reaction of the respective gallium halides in liquid ammonia at room temperature. Ga(NH_3_)_3_F_3_ was obtained from elemental gallium (purity≥99.9999 % trace metal basis) and NH_4_F in ammonia at 753 K and 150 MPa in a 97 mL autoclave made from the nickel‐based alloy Inconel 718® and equipped with a silver liner.^29^ The synthesis was carried out in an one‐sided closed tubular furnace LOBA 1200‐60‐400‐1 OW (HTM Reetz GmbH, Berlin, Germany), which produces a temperature gradient in the reaction vessel. Ga(NH_3_)_3_F_3_ crystallizes in the colder temperature zone of the autoclave. The pressure was monitored with a pressure transmitter and a digital analyzer (P2VA1/5000 bar and 1DA2510 by HBM, Darmstadt, Germany).

Samples for NMR measurements were prepared according to two different protocols. In case of samples intended for the identification of dissolved species, weighed amounts of GaCl_3_ (between 1 and 10 mg) and NH_4_Cl were filled in a 5 mm medium walled NMR tube and approximately 0.5 g of ammonia (Linde, purity≥99.999, and further purified with a MicroTorr MC400‐720F gas purifier, SAES Pure Gas, which reduces H_2_O, O_2_ and CO_2_ to <1 ppb) was condensed into the tubes via a self‐made Tensi‐Eudiometer after Hüttig.^30^ After filling, the ammonia was solidified by cooling with liquid nitrogen, the NMR tubes were evacuated and flame sealed. In most samples a solid residue remained, due to the low solubility of gallium halides in liquid ammonia. The volume of the resulting solution was determined from the measured fill height and the known inner diameter of the NMR tubes and used to calculate the concentration of NH_4_Cl in the sample. Samples to be used for quantization of dissolved gallium species were prepared in H‐shaped glass vessels allowing for direct decantation of a sample prepared in one leg into an NMR tube attached as second leg. Solutions were prepared by charging the vessels with known amounts of GaCl_3_, NH_4_Cl and NH_3_ (between 5 and 7 g). The mixture was allowed to equilibrate at room temperature and stirred for several minutes before part of the resulting saturated solution was decanted into the NMR tube. The liquids in both legs were then solidified by cooling with liquid nitrogen, and the NMR tube was flame sealed.

### NMR measurements

NMR spectra were recorded on a Bruker Avance AV 400 spectrometer (^1^H 400.1 MHz, ^71^Ga 122.0 MHz, ^81^Br 108.0 MHz, ^35^Cl 39.2 MHz, ^14^N 28.9 MHz) at ambient temperature (296–299 K) in unlocked mode if not stated otherwise. Chemical shifts were calibrated using the ^15^N signal of liquid ammonia (*δ*=−381.7 ppm at 298 K with a temperature dependence of 40 ppb^31^ as external standard and are referenced to external TMS using the Ξ‐scale^32^ employing TMS (^1^H, *Ξ*=100.000000 MHz), 1.1 m Ga(NO_3_)_3_ in D_2_O (^71^Ga, *Ξ*=30.496704 MHz), 0.01 m NaBr in D_2_O (^81^Br, *Ξ*=27.006518 MHz), 0.1 m NaCl in D_2_O (^35^Cl, *Ξ*=9.797909 MHz) and MeNO_2_ (^15^N, *Ξ*=10.136767 MHz; ^14^N, *Ξ*=7.226317 MHz) as secondary references. Measurements aiming at quantification of dissolved species were carried out using the FIXPUL method.^33^ All spectra were recorded using the same number of transients and processed by applying an exponential apodization function with a line broadening factor of 50 Hz prior to Fourier transformation. The signal strength was evaluated by both numerical integration and spectral deconvolution (with both methods yielding consistent results), and gallium concentrations were calculated as described^33^ from the measured signal integrals of the samples and a reference sample of known concentration (14.17(5) mm aqueous GaCl_3_).

### Computational studies

Density functional studies aiming at the calculation of molecular structures and electrostatic parameters (*q*_zz_) were carried out with the Gaussian 09^34^ suite of programs and a previously employed computational model^5^ based on the ωB97xD functional by Head‐Gordon^35^ and def2‐tzvp (geometry optimization) or def2‐tzvpp (calculation of *q*_zz_ at the final geometries) basis sets.^36^ Numerical integrations were performed on an ultrafine grid, and solvent effects were included by using a PCM model as implemented in the Gaussian package and employing the same solvent parameters for ammonia as in a previous study.^5^ Further details are listed in the ESI. Magnetic shieldings were then obtained for the final geometries by performing relativistic two‐component zero‐order regular approximation (ZORA) calculations including spin‐orbit coupling.^22^ These computations were carried out with the Amsterdam Density Functional package (ADF 2014)^37^ using an all‐electron, triple‐ζ, double‐polarization TZ2P Slater basis with the local density approximation (LDA) in the Vosko‐Wilk‐Nusair parameterization^38^ with nonlocal corrections for exchange (Becke88)^39^ and correlation (Perdew86)^40^ included in a self‐consistent manner. Chemical shifts were determined as δ_s_=(σ_ref_‐σ_s_)/(1‐σ_ref_) relative to [Ga(H_2_O)_6_]^3+^ for ^71^Ga using the magnetic shielding constant of [Ga(H_2_O)_6_]_3_^+^ (σ_ref_=1806.93 ppm) calculated at the same computational level as the reference.

The molecular dynamics simulations were performed in analogy to our previous work on [Ga(NH_2_)_4_]^−^ complexes in ammonia solution.^6^ To ensure best comparability, the same suite of force‐fields, time‐step (1 fs) and treatment of cut‐off (12 Å) potentials is used. However, to better accommodate dispersed ion solutions, our simulation cell was enlarged to 3000 ammonia molecules. After insertion of the discussed complexes, the models were relaxed at 300 K and 8 atm using the Nose‐Hover thermostat‐barostat combination.^6^ For sampling average solvation energies, we used 7.5 ns simulation runs of which the first 0.5 ns were truncated as initial relaxation. Proper convergence of sampling was ensured by monitoring the occurrence profiles of both volume and energy after relaxation and establishing Gaussian fits to the equilibrated data.

## Conflict of interest

The authors declare no conflict of interest.

## Supporting information

As a service to our authors and readers, this journal provides supporting information supplied by the authors. Such materials are peer reviewed and may be re‐organized for online delivery, but are not copy‐edited or typeset. Technical support issues arising from supporting information (other than missing files) should be addressed to the authors.

Supplementary
