# Supplementary material for: Approaching Dissolved Species in Ammonoacidic GaN Crystal Growth: A Combined Solution NMR and Computational Study
Source: Chemistry. 2020 Apr 28;26(31):7008–17. doi: 10.1002/chem.201904657 (PMC7317737; doi:10.1002/chem.201904657)
Supplement: Supplementary file 1 — Supplementary [file CHEM-26-7008-s001.pdf]

Chemistry–A European Journal

Supporting Information

# Approaching Dissolved Species in Ammonoacidic GaN Crystal Growth: A Combined Solution NMR and Computational Study

Peter Becker,[a] Tanakorn Wonglakhon,[b] Dirk Zahn,*[b] Dietrich Gudat,*[a] and Rainer Niewa*[a]

# Supporting Information

## Table of Contents


1. **NMR Spectra**

**Figure S1:** 122 MHz $^{71}$Ga NMR spectrum of a saturated solution of $GaCl_3$ in a solution of $NH_4Cl$ in liquid $NH_3$.

**Figure S2:** 39.2 MHz $^{35}$Cl and 108 MHz $^{81}$Br NMR spectrum of a solution of $GaCl_3$ and $NH_4Br$ in liq. $NH_3$.

2. **DFT Calculations**

**Details of Molecular Geometry Optimizations**

**Table S1:** Atomic coordinates and energies for complexes $[Ga(NH_3)_n]^{3+}$ ($n$ = 4 – 6), $[Ga(NH_3)_nX_{6-n}]^{(3-n)-}$ ($X$ = Cl, Br, I; $n$ = 1 – 3), $[Ga(NH_3)_nX_{6-n}]^{(n-3)+}$ ($X$ = Cl; $n$ = 4, 5), $[GaCl_n]^{(n-3)-}$ ($n$ = 5 – 6), $[Ga(NH_3)_{6-n}(NH_2)_n]^{(3-n)+}$ ($n$ = 1, 2) obtained after energy optimization.

**Table S2:** Atomic coordinates and energies for ion groups $\{[Ga(NH_3)_6]X_n\}^{(3-n)+}$ obtained after energy optimization.

**Figure S3:** Plot of calculated vs. observed $^{71}$Ga NMR chemical shifts for selected reference compounds.

**Figure S4:** Plot of calculated average Ga–N distances vs. calculated $^{71}$Ga NMR chemical shifts (left) and the number $n$ of chloride ligands (right) for aggregates $\{[Ga(NH_3)_6]Cl_n\}^{(3-n)+}$ ($n$ = 1 – 3), $\{[Ga(NH_3)_6]Cl_n\}^{(n-3)-}$ ($n$ = 4 – 6).

3. **MD Simulations**

**Details of Molecular Dynamics Simulations**

**Table S3:** Non-bonded interaction parameters

**Table S4:** Intra-molecular interaction parameters

4. **References**

## 1. NMR spectra

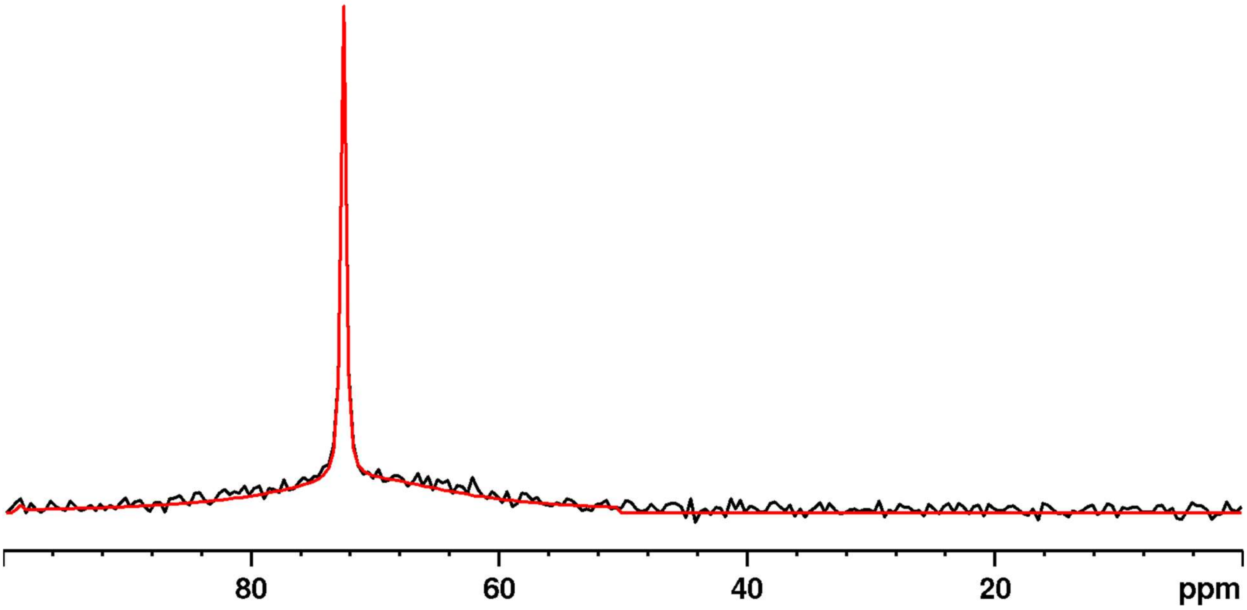


**Figure S1:** 122 MHz $^{71}$Ga NMR spectrum of a saturated solution of $GaCl_3$ (**4**) in a solution of $NH_4Cl$ (119mM) in liquid $NH_3$ with a sediment of undissolved salt at room temperature (black trace) and result of a fit (red trace) as two superimposed lines at $\delta^{71}$Ga = 72.7 ppm ($\Delta\nu_{½}$ = 52 Hz, 25 %) and 71.1 ppm ($\Delta\nu_{½}$ = 2190 Hz, 75 %). Note the different ratio of signal intensities compared to the spectrum of a sample prepared without mineralizer (see Figure 2).

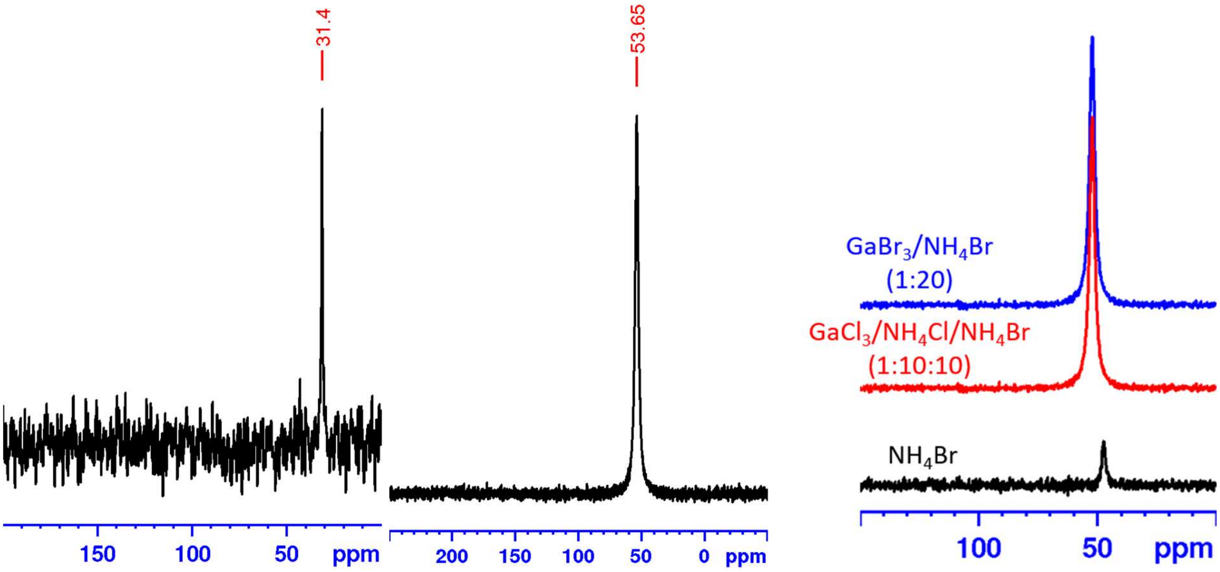


**Figure S2:** 39.2 MHz $^{35}$Cl (left) and 108 MHz $^{81}$Br NMR spectrum of a solution of $GaCl_3$ (9 mM) and $NH_4Br$ (190 mM) in liq. $NH_3$; comparison of the $^{81}$Br NMR spectra of solutions of $NH_4Br$, $GaCl_3/NH_4Br/NH_4Cl$ and $GaBr_3/NH_4Br$ in liq. $NH_3$ (right; numbers denote relative molar concentration of individual constituents)

## 2. DFT Calculations

**Molecular geometries**: Energy optimized molecular structures of complexes $[Ga(NH_3)_n]^{3+}$ ($n$ = 4 – 6), $[GaCl_n]^{(n-3)-}$ ($n$ = 5, 6), $[Ga(NH_3)_{6-n}(NH_2)_n]^{(3-n)+}$ ($n$ = 1 – 3), $[Ga(NH_3)_nX_{6-n}]^{(3-n)-}$ ($X$ = Cl, Br, I; $n$ = 1 – 3), $[Ga(NH_3)_nX_{6-n}]^{(n-3)+}$ ($X$ = Cl; $n$ = 4, 5), and $\{[Ga(NH_3)_5(NH_2)]Cl_2\}$ were computed with the Gaussian 09 program suite[S1] at the ωB97xD/def2-tzvp level. All calculations were performed using an ultrafine grid for numerical integrations and without any symmetry constraints. Energy optimization was first performed for isolated complexes/ion clusters (in the 'gas phase'), and the stationary states on the energy hypersurface found were verified as local minima by harmonic frequency calculations. The molecular structures were then re-optimized at the same computational level under inclusion of solvation effects as simulated by a PCM model (as implemented in the Gaussian package with the same solvent parameters for ammonia as in a previous study[S2]), and electrostatic parameters ($q_{zz}$) were computed at the resulting geometries at the PCM-ωB97xD/def2-tzvpp level. In some cases, the PCM-ωB97xD/def2-tzvp optimization runs ended in oscillations between closely similar structures and yielded a series of geometrically slightly different atomic configurations with very similar energies (fluctuations < 0.02 kcal/mol) instead of converging to a single, well-defined local minimum. Noticing that successful optimization to practically identical conformations was in some cases achievable by using a smaller basis set, we conclude that the convergence failure is presumably caused by numerical problems or inconsistencies in the modelling of the solvent cavity which prevent exact localization of a local minimum in very flat regions of the energy hypersurface. Single-point calculations on sets of 6 – 10 molecular configurations close to the energy threshold of individual optimization runs revealed that variations in computed energies and magnetic shieldings do not exceed values of 0.02 kcal/mol and 0.2 ppm, respectively, and we consider these results therefore as reasonable approximations to the properties of the 'true' local minimum structures.

**Table S1:** Atomic coordinates (in Å) and energies (in a.u.) for complexes $[Ga(NH_3)_n]^{3+}$ ($n$ = 4 – 6), $[Ga(NH_3)_nX_{6-n}]^{(3-n)-}$ ($X$ = Cl, Br, I; $n$ = 1 – 3), $[Ga(NH_3)_nX_{6-n}]^{(n-3)+}$ ($X$ = Cl; $n$ = 4, 5), $[GaCl_n]^{(n-3)-}$ ($n$ = 5 – 6), $[Ga(NH_3)_{6-n}(NH_2)_n]^{(3-n)+}$ ($n$ = 1, 2) obtained after energy optimization at the ωB97xD/def2-tzvpp (in the 'gas phase') or PCM-ωB97xD/def2-tzvpp level (in 'solution'), respectively.

| $[Ga(NH_3)_4]^{3+}$ gas phase<br>E = -2150.1674108 | $[Ga(NH_3)_4]^{3+}$ solution (liq. $NH_3$)*<br>E = -2150.820620 |
|---|---|
| Ga -0.0576741709 0.0077822871 0.075959847 | Ga -0.001009 0.000945 -0.000877 |
| N -1.9897871979 -0.3138848991 -0.3141913153 | N -1.658814 -0.056404 -1.042057 |
| N 1.013987129 -1.5675242359 -0.5229512107 | N -0.114546 -1.291928 1.461309 |
| N 0.1890582846 0.2733427844 2.0399726933 | N 0.252361 1.801467 0.726017 |
| N 0.5560320768 1.6392111467 -0.8990043876 | N 1.524524 -0.454565 -1.143099 |
| H 0.9358559064 -1.764534637 -1.527885993 | H -0.115695 -2.252281 1.119449 |
| H 2.0222297081 -1.4725076851 -0.3519779528 | H 0.668388 -1.215452 2.109790 |
| H 1.5449457996 1.8683310243 -0.7429108317 | H 2.413430 -0.379863 -0.649430 |
| H 0.4600492105 1.5732973182 -1.9194222251 | H 1.470461 -1.410756 -1.493868 |
| H -0.3364662244 1.0696594082 2.4201241429 | H 0.228669 2.517969 0.001176 |
| H -0.1056953694 -0.5303042614 2.6074971021 | H -0.470520 2.050132 1.400585 |
| H -2.3754119991 -1.1421119239 0.1550052692 | H -2.440319 0.353941 -0.531089 |
| H -2.6044957344 0.4583336601 -0.030250289 | H -1.594164 0.457010 -1.920941 |
| H 1.1630798393 0.4461807579 2.3160570109 | H 1.146004 1.903940 1.207148 |
| H -2.1933350791 -0.4583494152 -1.3104413751 | H -1.934847 -1.009451 -1.278367 |
| H 0.0454525044 2.4925427922 -0.6421988739 | H 1.599836 0.143908 -1.965044 |
| H 0.7516453165 -2.4470931213 -0.0621376109 | H -0.964636 -1.178383 2.012595 |

| $[Ga(NH_3)_5]^{3+}$ gas phase<br>E = -2206.8045188 | $[Ga(NH_3)_5]^{3+}$ solution (liq. $NH_3$)*<br>E = -2207.427356 |
|---|---|
| Ga 0.0002210523 0.0003013779 -0.0003269087 | Ga -0.001559 0.002601 -0.002363 |
| N 2.1409909554 -0.005252911 0.0115366494 | N -2.106693 0.003164 -0.018275 |
| N 0.0125277587 1.1301306558 -1.7094049563 | N 0.012323 -0.463887 1.947191 |
| N -2.1405140978 0.005816699 -0.0122699024 | N 2.102673 0.015449 -0.030374 |
| N -0.0076643624 0.9155360811 1.8326988091 | N -0.006670 1.934037 -0.561632 |
| N -0.0039756427 -2.044817951 -0.1240241274 | N 0.002307 -1.496639 -1.331153 |
| H 2.5578227516 -0.6094301528 0.7245465535 | H -2.506351 0.066836 -0.953509 |
| H 2.5647897243 0.9106713135 0.1807826336 | H -2.504292 0.786672 0.499623 |
| H 2.5673189087 -0.3203264 -0.8637490108 | H -2.513129 -0.831695 0.394699 |
| H -0.0055271127 1.9385471678 1.7696124755 | H -0.061361 2.552417 0.247231 |
| H 0.7985876727 0.6904752043 2.4210017234 | H -0.791157 2.183538 -1.157043 |
| H -0.820821829 0.6933186696 2.4125241487 | H 0.827300 2.211594 -1.074590 |
| H -0.8141859832 -2.4373728403 -0.6100733737 | H 0.780151 -2.138614 -1.207525 |
| H 0.8052368319 -2.440344157 -0.6093668799 | H -0.836617 -2.064846 -1.293597 |
| H -0.0052310878 -2.50162268 0.7935098796 | H 0.068631 -1.146850 -2.288116 |
| H 0.8250465317 1.7446630779 -1.8040476307 | H -0.825490 -0.171964 2.440817 |
| H 0.0142397488 0.564038378 -2.5638484387 | H 0.087787 -1.469210 2.086856 |
| H -0.7943464688 1.7508951453 -1.811436197 | H 0.792147 -0.054980 2.449670 |
| H -2.5586740163 -0.307242901 -0.8922089201 | H 2.512789 -0.833702 0.337206 |
| H -2.5614557165 0.9239432291 0.1520992446 | H 2.513663 0.766242 0.514304 |
| H -2.568349619 -0.5960310059 0.6961802284 | H 2.476677 0.119063 -0.963088 |

| $[Ga(NH_3)_6]^{3+}$ gas phase<br>E = -2263.4357328 | $[Ga(NH_3)_6]^{3+}$ solution (liq. $NH_3$)*<br>E = -2264.03314378 |
|---|---|
| Ga 0.0004052619 0.0001992492 0.0000052219 | Ga 0.006177 0.001309 -0.006586 |
| N 1.2144908331 -1.4198385012 1.0554394602 | N -1.581685 -0.778039 1.158396 |
| N -1.2150943169 1.4912963851 -0.9507233315 | N 1.668514 0.821315 -1.020888 |
| N -1.2456903965 -1.5374088383 -0.8297011315 | N -1.400969 0.683245 -1.424181 |
| N 1.2615551439 -0.2080178515 -1.7234224106 | N 0.159091 -1.767460 -1.146155 |

| | |
|---|---|
| N -1.263060269 0.0818390225 1.7326299846 | N -0.167614 1.847372 1.003314 |
| N 1.2481251521 1.5923102142 0.7159975855 | N 1.310292 -0.807085 1.445058 |
| H 0.8066700367 -2.3514918718 1.147063807 | H -1.703781 -1.783111 1.071426 |
| H 1.4172038003 -1.1427720809 2.0173727781 | H -2.487937 -0.378736 0.928625 |
| H 2.1333853622 -1.5877403798 0.6427171702 | H -1.461576 -0.611741 2.155109 |
| H -2.1676537908 -1.6201328788 -0.3983952942 | H -1.956807 1.469737 -1.098397 |
| H -0.8586682925 -2.4808736638 -0.7771471513 | H -2.074499 -0.036472 -1.675187 |
| H -1.4431686294 -1.4060458891 -1.8231347577 | H -0.986447 0.991545 -2.300639 |
| H 2.1785199963 0.2334524768 -1.6387325873 | H 1.039481 -2.261429 -1.028635 |
| H 1.4705120973 -1.1801545376 -1.9567655174 | H -0.564192 -2.448219 -0.926931 |
| H 0.8768841174 0.1766082638 -2.5875797254 | H 0.068886 -1.605282 -2.146537 |
| H -0.8714607376 0.588508114 2.5279995236 | H 0.530369 1.988852 1.727509 |
| H -1.4884352378 -0.8390164061 2.1126191824 | H -1.064545 1.970338 1.465020 |
| H -2.1724124562 0.5204182929 1.5789636848 | H -0.072111 2.644686 0.378903 |
| H 0.8475266432 2.1462488467 1.4745127431 | H 1.313866 -0.290000 2.320375 |
| H 2.1566057208 1.294907226 1.075319899 | H 1.053638 -1.759534 1.692390 |
| H 1.4747965031 2.2828010646 -0.001641707 | H 2.285004 -0.856027 1.160241 |
| H -1.4269264643 2.2864038588 -0.3456693927 | H 2.301088 1.317104 -0.396089 |
| H -2.1297463499 1.1619915208 -1.2635271325 | H 1.434545 1.492483 -1.747669 |
| H -0.8035247275 1.9164633633 -1.7829039014 | H 2.240133 0.119784 -1.484162 |

| $[Ga(NH_3)_5Br]^{2+}$ gas phase<br>E = -4781.5945777 | $[Ga(NH_3)_5Br]^{2+}$ solution (liq. $NH_3$)*<br>E = -4781.88346 |
|---|---|
| Ga 2.9672525572 1.9068736932 1.4243334504 | Ga -0.552197 0.000576 -0.002931 |
| N 4.0040707542 1.8527335671 3.3251485702 | N -2.675489 -0.047434 -0.019147 |
| N 1.6817392215 3.4001993975 2.3355214675 | N -0.612306 -2.019296 -0.627590 |
| N 4.3310723995 0.3831854365 0.701501787 | N -0.612468 2.017939 0.636604 |
| N 4.3618305476 3.4245394782 0.7579001602 | N -0.520649 0.681740 -1.998648 |
| N 1.6581723943 0.3217865109 2.1108807597 | N -0.529734 -0.587365 2.025329 |
| H 4.8619829177 1.3049777819 3.3159604121 | H -3.102588 0.838553 0.236686 |
| H 3.4430036925 1.4615803527 4.0790016479 | H -3.057513 -0.733153 0.624795 |
| H 4.2776499781 2.7777623379 3.6502257038 | H -3.057845 -0.286953 -0.929685 |
| H 4.313993689 -0.5174668678 1.1750973113 | H -1.061212 2.164109 1.536685 |
| H 5.3143717159 0.6369520559 0.6335181413 | H -1.066710 2.657623 -0.009256 |
| H 4.0242244735 0.202099049 -0.2547855825 | H 0.346858 2.340942 0.737724 |
| H 3.8878764876 4.3195591118 0.6508609549 | H -0.250034 -0.051025 -2.647763 |
| H 5.2303460338 3.6198158288 1.2514934089 | H -1.378794 1.087653 -2.362212 |
| H 4.6193088838 3.1740655678 -0.1959850458 | H 0.199531 1.393886 -2.083882 |
| H 0.7560663376 0.674904374 2.4240769035 | H -0.296490 -1.569397 2.138771 |
| H 1.9566583181 -0.3478270897 2.8169178389 | H -1.380092 -0.430995 2.559600 |
| H 1.4354257364 -0.2163440726 1.2738217881 | H 0.213558 -0.079017 2.497437 |
| H 1.202752706 3.1526110959 3.1986641728 | H -1.149030 -2.636233 -0.023583 |
| H 2.1009858381 4.3102196569 2.5140710385 | H -0.983606 -2.162754 -1.562561 |
| H 0.9526914198 3.565651429 1.6409057037 | H 0.340588 -2.374406 -0.637323 |
| Br 1.8139838978 2.0276773052 -0.6341285923 | Br 1.927314 -0.014165 -0.002869 |

| $[Ga(NH_3)_5Br]^{2+}$ gas phase<br>E = -2667.5905834 | $[Ga(NH_3)_5Cl]^{2+}$ solution (liq. $NH_3$)*<br>E = -2667.879528 |
|---|---|
| Ga 2.9639523431 1.896299883 1.4508708269 | Ga 0.167186 -0.006975 0.000606 |
| N 4.0369050144 1.8877837216 3.3203217542 | N 2.287476 -0.036259 -0.012298 |
| N 1.7521678522 3.4908947888 2.269323594 | N 0.207103 -1.657528 -1.326068 |
| N 4.2441417324 0.3125735967 0.7184656534 | N 0.193143 1.624449 1.356170 |
| N 4.3764270711 3.3255050595 0.6536468563 | N 0.123333 -1.322404 1.657333 |
| N 1.6298272197 0.3882231019 2.235023563 | N 0.220122 1.393839 -1.584109 |
| H 4.5458169559 2.7524192764 3.4929207762 | H 2.678207 -0.872565 0.411600 |
| H 4.7300957722 1.1463185418 3.3981311858 | H 2.696616 0.747941 0.487342 |
| H 3.4251178133 1.7719845896 4.1257701008 | H 2.670283 0.003066 -0.952783 |
| H 4.1809987072 -0.5894383462 1.1854433556 | H 0.603262 2.475693 0.984123 |
| H 5.2395427856 0.5084362095 0.6390654095 | H 0.660109 1.451923 2.241532 |
| H 3.9134107141 0.1550475048 -0.2341169308 | H -0.774475 1.843998 1.578120 |

| | |
|---|---|
| H 4.1246458382 4.2986132766 0.8136993752 | H 0.141691 -2.303772 1.396521 |
| H 5.3649038317 3.2558898638 0.8852545464 | H 0.855572 -1.202884 2.350920 |
| H 4.3036193177 3.2069165652 -0.3569988202 | H -0.762535 -1.183233 2.136244 |
| H 0.7626817512 0.7736547236 2.6033978844 | H 0.260168 0.974185 -2.507681 |
| H 1.9490041855 -0.2868347336 2.926479401 | H 0.976635 2.070833 -1.549090 |
| H 1.3359514111 -0.1453543176 1.4170476635 | H -0.650686 1.916975 -1.557525 |
| H 1.1383751387 3.2547032397 3.0458177178 | H 0.256459 -1.383792 -2.302591 |
| H 2.222689973 4.3469218617 2.5543710387 | H 0.957530 -2.328551 -1.187881 |
| H 1.1362685537 3.7487250239 1.4973534531 | H -0.668479 -2.163539 -1.224789 |
| Cl 1.8189160181 1.9362725692 -0.4462864049 | Cl -2.135374 0.009133 -0.056473 |

| $[Ga(NH_3)_5I]^{2+}$ gas phase<br>E = -2505.1588082 | $[Ga(NH_3)_5I]^{2+}$ solution (liq. $NH_3$)*<br>E = -2505.44605395 |
|---|---|
| Ga 2.9367427905 1.8947257726 1.3902064317 | Ga -0.896446 0.000488 0.000404 |
| N 4.0574554157 1.7502728411 3.2537972452 | N -3.026611 -0.045597 -0.034228 |
| N 1.7595858193 3.458207765 2.3511185233 | N -0.990031 2.049429 0.539525 |
| N 4.3049538079 0.3479713065 0.6785716875 | N -0.980622 -2.053010 -0.506753 |
| N 4.3233366456 3.4388961908 0.7263375545 | N -0.880823 -0.486166 2.055185 |
| N 1.5777843226 0.3998995862 2.205920779 | N -0.880717 0.565665 -2.029411 |
| H 5.0016163705 2.1248159028 3.1916363511 | H -3.428766 -0.588743 0.724066 |
| H 4.1743103889 0.7885724791 3.5656770038 | H -3.390843 -0.460929 -0.886988 |
| H 3.6264803253 2.2370414435 4.0367896759 | H -3.453877 0.873765 0.028589 |
| H 4.1583550422 -0.584928236 1.0582033191 | H -1.323068 -2.237441 -1.445513 |
| H 5.3042676858 0.5206916238 0.7652796446 | H -1.559244 -2.611581 0.114333 |
| H 4.1132248052 0.2675335067 -0.3202001992 | H -0.042451 -2.442773 -0.463631 |
| H 3.7535917638 4.130113385 0.2397647503 | H -0.193372 0.093912 2.528673 |
| H 4.9141385714 3.9556572024 1.3746406108 | H -1.758237 -0.370325 2.554731 |
| H 4.9437346998 3.0807999402 0.0025682715 | H -0.580222 -1.443126 2.216790 |
| H 0.6323137683 0.7478835056 2.0528259934 | H -0.270192 1.368358 -2.153368 |
| H 1.6183822217 0.0905075073 3.1750161231 | H -1.778871 0.814064 -2.434700 |
| H 1.6186540768 -0.4418440502 1.6338603492 | H -0.484614 -0.162454 -2.616908 |
| H 1.183681161 3.1773619304 3.1419607295 | H -1.450185 2.648776 -0.140026 |
| H 2.2598754437 4.2896170451 2.6583378578 | H -1.452818 2.226407 1.426788 |
| H 1.1070880344 3.7725173522 1.6324756315 | H -0.040446 2.400656 0.634976 |
| I 1.6258868397 1.989242 -0.8197863337 | I 1.817145 -0.006338 -0.005105 |

| cis-$[Ga(NH_3)_4Cl_2]^+$ gas phase<br>E = -3071.7093931-3071.6055859 | cis-$[Ga(NH_3)_4Cl_2]^+$ solution (liq. $NH_3$)<br>E = -3071.7093931 |
|---|---|
| Ga -0.4311629286 0.0010387347 0.0720756312 | Ga -0.3948154466 0.0010697889 0.0959303039 |
| Cl -1.6024549583 1.7795482931 -0.6499816713 | Cl -1.7142991142 1.7425754744 -0.7039305917 |
| Cl -1.5630225072 -1.771907393 -0.7228277893 | Cl -1.6568085371 -1.7339675609 -0.8045194159 |
| N 0.9385716257 -1.4819373411 0.9801105288 | N 0.8819020581 -1.4831509011 0.947664945 |
| N 0.7732429209 0.034769407 -1.717706222 | N 0.7851717629 0.0490461835 -1.6547465642 |
| N 0.9492116859 1.4773817576 0.9751354071 | N 0.8972738157 1.4784998685 0.9360892277 |
| N -1.5998933614 -0.0326729595 1.88521755 | N -1.5374490591 -0.0468339519 1.871220838 |
| H 1.2319808206 -1.3597028082 1.943859895 | H 1.2278206853 -1.2876115288 1.8812840457 |
| H 1.7799296314 -1.6548713317 0.4390724625 | H 1.6942491599 -1.6944873051 0.3773365519 |
| H 0.3879176147 -2.3362206557 0.9256461461 | H 0.3398462166 -2.3405180601 1.0001094762 |
| H 1.9500492262 1.3521118132 0.8634339919 | H 1.8908665804 1.2793131755 0.8846872069 |
| H 0.7853943288 1.648621089 1.9622259049 | H 0.6949465594 1.6881603582 1.9081724436 |
| H 0.6843592933 2.3336637958 0.4928246097 | H 0.7346372765 2.337780127 0.4195247066 |
| H -2.2824725449 -0.7664301336 1.7063142452 | H -2.2385598086 -0.7726650118 1.7555228641 |
| H -1.2138268333 -0.1948026799 2.8092100285 | H -1.0572260743 -0.2295149829 2.7465116619 |
| H -2.1166898078 0.8430540171 1.9071005816 | H -2.0357760159 0.8320823887 1.9726134799 |
| H 0.5854039694 -0.8388455878 -2.2034760657 | H 0.6778398861 -0.8280334247 -2.1550621959 |
| H 1.7753037244 0.19247588 -1.7275386894 | H 1.7797199683 0.2272947956 -1.5587338493 |
| H 0.3425151004 0.7719121029 -2.2721475449 | H 0.4050170865 0.7781465672 -2.2511261343 |

| trans-$[Ga(NH_3)_4Cl_2]^+$ gas phase<br>E = -3071.7093931-3071.6193777 | trans-$[Ga(NH_3)_4Cl_2]^+$ solution (liq. $NH_3$)<br>E = -3071.7093931-3071.7133485 |
|---|---|

| | |
|---|---|
| Ga -0.0000032219 -0.000016337 0.0001338993 | Ga 0.0004613096 -0.0028429387 -0.0018591228 |
| Cl 2.2601475314 0.00015536 0.0002742822 | Cl 2.336337789 -0.0053917436 -0.005464263 |
| Cl -2.2601538071 -0.0001880826 -0.0000065151 | Cl -2.3360422971 -0.0045679756 -0.0001618545 |
| N 0.0435222341 0.4841123707 -2.1073537314 | N 0.0355594428 -1.1081870439 -1.8006214564 |
| N -0.0436246444 2.107470696 0.484256719 | N -0.0411533038 1.7956997365 -1.1068180986 |
| N 0.0433341433 -0.4841388471 2.1076267174 | N 0.0410829345 1.1091125641 1.7940479254 |
| N -0.0432441991 -2.1075097481 -0.4839945137 | N -0.0353681528 -1.7979194564 1.1094130546 |
| H 0.5501149193 0.2179707185 2.6376969532 | H 0.5559224964 1.9777728922 1.6897915738 |
| H 0.5392159645 -1.3535719247 2.2776462136 | H 0.5009697395 0.6036017535 2.5442287543 |
| H -0.8916384602 -0.5674068667 2.4969600997 | H -0.8964758043 1.340122534 2.1079073724 |
| H -0.539002462 -2.2775360761 -1.3534967137 | H -0.4987304836 -2.5474464929 0.6062606302 |
| H 0.8917453681 -2.4968302027 -0.5671323877 | H 0.903585841 -2.1120644626 1.3344313414 |
| H -0.5501154825 -2.6375870003 0.2180444206 | H -0.5450935747 -1.6932472962 1.9806204274 |
| H -0.5504893837 2.6374706391 -0.2178453029 | H -0.5038000933 1.6844346102 -2.0032859952 |
| H 0.8912954087 2.4969333034 0.5675105132 | H 0.8955336631 2.1442785093 -1.2845171229 |
| H -0.5395167244 2.2774219641 1.3536972785 | H -0.5555376237 2.5218619906 -0.6191217184 |
| H 0.5392908202 1.3536220384 -2.2773117223 | H 0.5208771125 -0.6099589959 -2.5394659058 |
| H 0.5504776599 -0.2179190142 -2.6373605124 | H 0.5235405105 -1.9918424708 -1.6960509808 |
| H -0.8914146641 0.5672360091 -2.4968036974 | H -0.9039105054 -1.3105717136 -2.1287475611 |

| cis-$[Ga(NH_3)_4Cl_2]^+$ gas phase<br>E = -7299.6139368 | cis-$[Ga(NH_3)_4Br_2]^+$ solution (liq. $NH_3$)<br>E = -7299.7185352 |
|---|---|
| Ga -0.4637735134 0.0037591231 0.0816844077 | Ga -0.3985035653 -0.0077077243 0.1199567326 |
| N 0.9424992497 -1.478431506 0.9545972187 | Br -1.7577695665 -1.8805692685 -0.8285798498 |
| N -1.5559063716 0.0554650939 1.9511199145 | Br -1.8580739634 1.8466693781 -0.7284678591 |
| N 0.7354701822 -0.0471176147 -1.7208246822 | N 0.8921692025 -1.4959496109 0.9400152286 |
| N 0.944789109 1.4773292222 0.9651659198 | N -1.5105051219 -0.0483302246 1.9126898586 |
| Br -1.6773249989 1.9058560302 -0.756418607 | N 0.7573062965 0.0734205984 -1.6430431661 |
| Br -1.7570253252 -1.8915775161 -0.6448496629 | N 0.8906245665 1.4861955139 0.9299719766 |
| H 0.714268264 -1.7207433118 1.9138685399 | H 1.4976411637 -1.2033950284 1.7008999716 |
| H 1.9420285491 -1.3034263662 0.9264388676 | H 1.4917438485 -1.9100491925 0.2326932875 |
| H 0.7534195308 -2.316551747 0.4089790518 | H 0.3180846758 -2.2565551618 1.291223253 |
| H 1.7143743007 1.7104210843 0.3451685586 | H 1.8822085398 1.2708631266 0.9059522497 |
| H 1.348429694 1.3020070719 1.8799300486 | H 0.672999681 1.7302206903 1.8909862519 |
| H 0.3780443861 2.3205047537 1.0269653276 | H 0.754960835 2.3343958898 0.3875326437 |
| H -2.0385588107 -0.8349925803 2.0437063161 | H -2.1962708766 -0.794218318 1.8351978323 |
| H -1.1240171078 0.2763409403 2.8424736703 | H -1.0054286599 -0.19157862 2.7818245003 |
| H -2.2746057696 0.7563377688 1.7807953331 | H -2.0303564442 0.8200190305 1.9999429395 |
| H 0.2590165455 -0.7390375661 -2.2961714886 | H 0.5511919066 -0.7420796216 -2.2124196759 |
| H 1.7233073064 -0.2794852688 -1.7255278619 | H 1.7661973813 0.1309169706 -1.5454027786 |
| H 0.6169437798 0.8476533885 -2.1895698718 | H 0.4575841006 0.8853835727 -2.1752633963 |

| trans-$[Ga(NH_3)_4Br_2]^+$ gas phase<br>E = -7299.6278641 | trans-$[Ga(NH_3)_4Br_2]^+$ solution (liq. $NH_3$)<br>E = -7299.7183556 |
|---|---|
| Ga 0.6215154574 0.0082184236 0.0005270464 | Ga -0.3992975935 0.0292035187 0.1242755005 |
| N -0.9021949948 -0.1577837638 -1.5270304242 | N 0.8722403335 -1.4846822053 0.9233220788 |
| N -0.9128718235 0.0688733691 1.5252881691 | N -1.5132386542 -0.0263732141 1.9137764349 |
| N 2.1529603439 0.0403762999 -1.5278588357 | N 0.7118090685 0.035347794 -1.6641885416 |
| N 2.1477955808 0.0814531986 1.532688506 | N 0.9599243065 1.4478714488 0.9545050878 |
| Br 0.7048995785 -2.4160879699 0.10691488 | Br -1.7735534757 1.9641781802 -0.6887280129 |
| Br 0.5379557122 2.432566586 -0.105966553 | Br -1.8538459738 -1.7710008555 -0.8307223755 |
| H 2.9875547394 -0.4082778841 1.2390101583 | H 1.1336855632 -1.3461809763 1.8941994823 |
| H 1.845068433 -0.3949373216 2.3768875281 | H 1.7388870132 -1.6126953359 0.4098178828 |
| H 2.4003117798 1.035862317 1.7752322945 | H 0.3721675542 -2.3674447029 0.8708063454 |
| H -1.7522511745 0.5281194758 1.1849965932 | H 1.9421631716 1.245225947 0.7977698184 |
| H -1.1646643779 -0.8605885637 1.8511688088 | H 0.8551344819 1.5818709621 1.9551499021 |
| H -0.6153533466 0.620000366 2.324671202 | H 0.7649110331 2.3467326471 0.5227990124 |
| H 1.8213168119 0.4982756323 -2.3714257033 | H -2.0192533233 -0.9060682674 1.9576253632 |
| H 2.4686381397 -0.8946014398 -1.7721403062 | H -1.0201752485 0.0846318037 2.79388392 |
| H 2.9578869034 0.5837278746 -1.2310183826 | H -2.2088276996 0.7125175989 1.8623438999 |

| | |
|---|---|
| H -0.5648014572 -0.6826328939 -2.3282501077 | H 0.7891219867 -0.9028649677 -2.0446191433 |
| H -1.7062656478 -0.6780209556 -1.1892263802 | H 1.645992422 0.4306178958 -1.6274105053 |
| H -1.2202766578 0.7521482494 -1.8500314935 | H 0.1835320342 0.5834237288 -2.33707515 |

| cis-$[Ga(NH_3)_4I_2]^+$ gas phase<br>E = -2746.7413833 | cis-$[Ga(NH_3)_4I_2]^+$ solution (liq. $NH_3$)<br>E = -2746.8454221 |
|---|---|
| Ga -0.5113608767 -0.0346904893 0.0745615959 | Ga -0.4056161935 -0.0080497533 0.114377839 |
| N 0.9647775343 -1.4659119846 0.9815290191 | N 0.8897921015 -1.4874950941 0.9393161609 |
| N -1.5027977495 0.0769691753 2.0172144209 | N -1.5041322717 -0.0499302737 1.9246143853 |
| N 0.7017569091 -0.0307080374 -1.7414092913 | N 0.7722252101 0.0741584588 -1.642139458 |
| N 0.9240873161 1.4837673763 0.8729896827 | N 0.8809875112 1.4882259958 0.9240385899 |
| I -1.9598428615 1.960834237 -0.8322447125 | I -2.0241208188 2.0023454847 -0.8240938836 |
| I -1.8327270435 -2.1827993088 -0.638983853 | I -1.8835117353 -2.0742725741 -0.9236098886 |
| H 0.4421439269 -2.0576698514 1.6213246353 | H 1.5136437418 -1.175868558 1.6780901416 |
| H 1.8029263285 -1.1563124638 1.4648173899 | H 1.4729520552 -1.9180241155 0.2277651388 |
| H 1.2536541413 -2.0967964535 0.2391053762 | H 0.3266811654 -2.2411951565 1.3222695222 |
| H 1.8588630648 1.4692977054 0.4773191008 | H 1.8701158796 1.2588719695 0.9217735065 |
| H 1.0348863536 1.5096969958 1.8817618672 | H 0.6477146207 1.7404528628 1.8794433418 |
| H 0.4978178198 2.3670390639 0.5975082532 | H 0.771387435 2.3369820967 0.3762489773 |
| H -2.3055802479 -0.5398451611 1.9088174701 | H -2.2397218822 -0.7441900846 1.8241575325 |
| H -1.0378791372 -0.1864328735 2.880513921 | H -0.9922041417 -0.2770352109 2.7723204991 |
| H -1.8762440348 1.0158074575 2.1323589593 | H -1.9650219419 0.842714832 2.0767223154 |
| H 0.1994580525 -0.6618285957 -2.3627356691 | H 0.5711558395 -0.7371227184 -2.2199206738 |
| H 1.6751328041 -0.3193955833 -1.7452989255 | H 1.7801803175 0.1231342339 -1.5268479048 |
| H 0.6437107001 0.8928727912 -2.1630322403 | H 0.4902761076 0.8901916049 -2.1784091415 |

| trans-$[Ga(NH_3)_4I_2]^+$ gas phase<br>E = -2746.754378 | trans-$[Ga(NH_3)_4I_2]^+$ solution (liq. $NH_3$)<br>E = -2746.754378 |
|---|---|
| Ga 0.6217714129 0.0080513358 0.0007824087 | Ga 0.000214 0.000191 0.000003 |
| N -0.9073984896 -0.1597468128 -1.5325714982 | N -0.060524 1.466310 -1.512473 |
| N -0.918016748 0.0696767763 1.5314430882 | N 0.060386 1.513381 1.465388 |
| N 2.1580995228 0.0467770836 -1.5340804139 | N 0.060237 -1.512547 -1.465802 |
| N 2.1545234412 0.0755792342 1.5382141559 | N -0.060373 -1.465317 1.513010 |
| I 0.7135028994 -2.647467794 0.1127620835 | I -2.762310 -0.000238 0.000002 |
| I 0.5299721563 2.663593503 -0.111181782 | I 2.762256 -0.000235 -0.000025 |
| H 2.9920873648 -0.4162474567 1.2407185342 | H -0.549340 -2.301718 1.207958 |
| H 1.8495568101 -0.4051525943 2.3795173751 | H -0.561039 -1.137651 2.333658 |
| H 2.4114040854 1.0277327952 1.7868538168 | H 0.871558 -1.743286 1.809342 |
| H -1.7551887055 0.5320700974 1.189039571 | H 0.561475 2.333805 1.137804 |
| H -1.1743231725 -0.8581101277 1.8599609984 | H -0.871743 1.810018 1.742376 |
| H -0.6177898792 0.6218780657 2.329471249 | H 0.548538 1.208630 2.302375 |
| H 1.8228551686 0.5079253163 -2.3748308371 | H 0.548902 -1.207513 -2.302399 |
| H 2.4787011858 -0.8854653506 -1.7841764505 | H -0.871854 -1.808701 -1.743437 |
| H 2.9603937827 0.5929476489 -1.2342163441 | H 0.560954 -2.333298 -1.138448 |
| H -0.5676690077 -0.6878515797 -2.3310636393 | H -0.561871 1.139300 -2.332968 |
| H -1.7101757493 -0.6809478788 -1.1922632747 | H -0.548819 2.302900 -1.206798 |
| H -1.2282190781 0.7481397381 -1.860023041 | H 0.871385 1.743903 -1.809229 |

| fac-$[Ga(NH_3)_3Br_3]$ gas phase<br>E = -9817.4987945 | fac-$[Ga(NH_3)_3Br_3]$ solution (liq. $NH_3$)<br>E = -9817.5411496 |
|---|---|
| Ga -0.0012766509 0.0050784231 0.0861458721 | Ga 0.0006056675 0.0063593458 0.2067067461 |
| N -0.9388701971 1.4687870825 1.5068687485 | N -0.910579497 1.4521189826 1.4927278239 |
| N -0.802267777 -1.542087699 1.5009584625 | N -0.8009648038 -1.5086982035 1.4800238289 |
| N 1.7372717116 0.0802102137 1.5045822578 | N 1.7062050795 0.0632456461 1.4914525148 |
| Br -2.1939239498 -0.0649542607 -0.9892293358 | Br -2.1577378557 -0.0196286409 -1.0883402234 |
| Br 1.1572567088 -1.8542294562 -0.9939245143 | Br 1.1025250608 -1.8442480441 -1.0926503157 |
| Br 1.0315454989 1.9418379721 -0.9859133316 | Br 1.0572356821 1.8934583596 -1.0795889162 |
| H -0.7564131718 1.4813346809 2.5028842685 | H -0.5692402996 1.5095157808 2.4463148477 |
| H -1.9377464745 1.3841560087 1.346809427 | H -1.9130385113 1.2989819422 1.5305900933 |
| H -0.6394867709 2.3551534817 1.1098366927 | H -0.7641158524 2.3605188161 1.0633830205 |

| | |
|---|---|
| H -1.7169247636 -1.7265228158 1.0979772054 | H -1.6622592936 -1.8281003398 1.0470262589 |
| H -0.911249568 -1.3897347721 2.496150073 | H -1.0222585201 -1.2496887193 2.4359369943 |
| H -0.2281575636 -2.3646454541 1.3454125057 | H -0.1710295175 -2.3038171801 1.5110789837 |
| H 2.1649117681 0.987314312 1.3471900947 | H 2.0985811103 0.9991494962 1.5015326507 |
| H 1.6571928936 -0.0867764535 2.5001751937 | H 1.575859736 -0.2290096983 2.4544487478 |
| H 2.3536403064 -0.6222102632 1.1050623799 | H 2.4057138147 -0.5474465434 1.0803429447 |

| fac-[Ga(NH$_3$)$_3$I$_3$] gas phase<br>E = -2988.1936273 | fac-[Ga(NH$_3$)$_3$I$_3$] solution (liq. NH$_3$)*<br>E = -2988.23450187 |
|---|---|
| Ga -0.0005597584 0.003329408 0.0739407575 | Ga -0.003531 -0.001383 0.843132 |
| N -0.9242707739 1.4704087625 1.5219631527 | N -1.187564 -1.225923 2.135274 |
| N -0.812522529 -1.529842302 1.5209805528 | N 1.644566 -0.411812 2.143546 |
| N 1.7303758164 0.0685663079 1.5244593459 | N -0.472941 1.645559 2.119695 |
| I -2.4003013761 -0.0266382671 -1.1037650924 | I 0.589562 -2.290880 -0.578035 |
| I 1.228266721 -2.0604267332 -1.0998351566 | I 1.697586 1.652560 -0.575890 |
| I 1.1752621727 2.0988765616 -1.0977974508 | I -2.282155 0.637547 -0.579501 |
| H -0.6046587263 1.552923032 2.4801781541 | H -1.334793 -0.884678 3.079920 |
| H -1.9276275344 1.3168963379 1.5115143246 | H -0.788480 -2.157779 2.202845 |
| H -0.7484578222 2.3557993386 1.0538078352 | H -2.102865 -1.327551 1.707289 |
| H -1.6672429436 -1.8190741804 1.0522043412 | H 2.176016 -1.173964 1.733077 |
| H -1.0440174141 -1.2949900121 2.4793003153 | H 1.419848 -0.679896 3.096268 |
| H -0.1787084548 -2.3226809977 1.5102170934 | H 2.265733 0.390736 2.184931 |
| H 2.0969700486 1.0151417253 1.5160840912 | H -1.472365 1.710909 2.287312 |
| H 1.6419711933 -0.2517046548 2.4819215865 | H -0.005979 1.657842 3.021645 |
| H 2.4110233808 -0.5238733264 1.0558121495 | H -0.200740 2.493456 1.631531 |

| mer-[Ga(NH$_3$)$_3$Br$_3$] gas phase<br>E = -9817.5452477 | mer-[Ga(NH$_3$)$_3$Br$_3$] solution (liq. NH$_3$)<br>E = -9817.5452477 |
|---|---|
| Ga 0.0037 -0.03942 0.00001 | Ga 0.0153995966 -0.063468946 -0.0001925978 |
| Br -2.47218 -0.44004 0.00011 | Br -2.5343403354 -0.247739543 0.0004989975 |
| Br 2.48712 -0.34554 0.00006 | Br 2.5646642337 -0.2562250807 -0.0007521432 |
| Br -0.04717 2.41641 -0.00022 | Br 0.0302620888 2.444589398 -0.0012477939 |
| N 0.06575 -2.28161 0. | N 0.0445024553 -2.1924489735 0.0007342335 |
| N -0.01497 -0.03962 -2.13571 | N -0.0137434968 -0.085173262 -2.0917154276 |
| N -0.01491 -0.03922 2.13573 | N -0.0126408135 -0.0835919445 2.0913623482 |
| H -0.87724 -2.65411 -0.00157 | H -0.8972971472 -2.5721494376 0.0011924351 |
| H 0.57025 -2.61868 0.81082 | H 0.5317844144 -2.5594784133 0.8114755596 |
| H 0.57323 -2.6186 -0.80898 | H 0.5313260527 -2.5602581514 -0.8099306333 |
| H -0.15546 0.94551 2.34195 | H -0.2794326259 0.8437456812 2.4069454085 |
| H -0.78189 -0.56367 2.53898 | H -0.682378087 -0.7412026113 2.4770339804 |
| H 0.8719 -0.32682 2.53296 | H 0.9049745169 -0.287724055 2.4751438129 |
| H -0.15585 0.94501 -2.34212 | H -0.2825633205 0.8413917892 -2.4078404574 |
| H -0.78178 -0.56441 -2.53885 | H -0.6822511981 -0.7444175755 -2.4767235559 |
| H 0.87192 -0.32703 -2.53288 | H 0.9041516661 -0.2876828746 -2.4756931663 |

| mer-[Ga(NH$_3$)$_3$Cl$_3$] solution (liq. NH$_3$)<br>E = -3475.5292319 | mer-[Ga(NH$_3$)$_3$Cl$_3$] solution (liq. NH$_3$)<br>E = -3475.5292319 |
|---|---|
| Ga 0.0037 -0.03942 0.00001 | Ga 0.0077062657 -0.0610062757 0.0080274347 |
| Cl -2.2945 -0.41129 0.0001 | Cl -2.3583963233 -0.2742729852 0.0392086157 |
| Cl 2.30847 -0.32352 0.00006 | Cl 2.376257214 -0.204480373 -0.0063341643 |
| Cl -0.04344 2.23645 -0.0002 | Cl -0.0211419091 2.2771511309 -0.0079535357 |
| N 0.06575 -2.28161 0. | N 0.0593529804 -2.1982156076 -0.018953105 |
| N -0.01491 -0.03922 2.13573 | N 0.0078593591 -0.0722636019 2.104120521 |
| N -0.01497 -0.03962 -2.13571 | N -0.0334700245 -0.0934201347 -2.0888373799 |
| H -0.87724 -2.65411 -0.00157 | H -0.8582959814 -2.5792781752 -0.2246341577 |
| H 0.57025 -2.61868 0.81082 | H 0.3614425428 -2.5890001287 0.8667823209 |
| H 0.57323 -2.6186 -0.80898 | H 0.7136261184 -2.5364249321 -0.7161077988 |
| H -0.15546 0.94551 2.34195 | H -0.1658995853 0.8803581467 2.408083699 |
| H -0.78189 -0.56367 2.53898 | H -0.7196237012 -0.6570723304 2.5011060339 |
| H 0.8719 -0.32682 2.53296 | H 0.9040012878 -0.3576077148 2.4845913155 |

| | |
|---|---|
| H -0.15585 0.94501 -2.34212 | H -0.2169286756 0.854085243 -2.4026456582 |
| H -0.78178 -0.56441 -2.53885 | H -0.7662432876 -0.6872471856 -2.4616510206 |
| H 0.87192 -0.32703 -2.53288 | H 0.8549397197 -0.3823280759 -2.4845071204 |
| mer-$[Ga(NH_3)_3Br_3]$ gas phase | mer-$[Ga(NH_3)_3I_3]$ solution (liq. $NH_3$) |
| E = -2988.2377071 | E = -2988.2377071 |
| Ga 0.0037 -0.03942 0.00001 | Ga 0.0150946361 -0.0561647549 -0.000366487 |
| I -2.68936 -0.47518 0.00012 | I -2.7881454335 -0.255002392 0.0006519207 |
| I 2.70547 -0.37246 0.00007 | I 2.8194595054 -0.2725642661 -0.0002321819 |
| I -0.05111 2.60637 -0.00023 | I 0.0384922472 2.6779344382 -0.0032548321 |
| N 0.06575 -2.28161 0. | N 0.0503838731 -2.1810025495 0.0013245217 |
| N -0.01497 -0.03962 -2.13571 | N -0.0183785796 -0.0990779193 -2.098019661 |
| N -0.01491 -0.03922 2.13573 | N -0.0173893777 -0.0944104533 2.0973859608 |
| H -0.87724 -2.65411 -0.00157 | H -0.8898037488 -2.5667406716 0.0009142238 |
| H 0.57025 -2.61868 0.81082 | H 0.5382455087 -2.5476457052 0.812480963 |
| H 0.57323 -2.6186 -0.80898 | H 0.5395861117 -2.5488278782 -0.8084937325 |
| H -0.15546 0.94551 2.34195 | H -0.2974057223 0.8245088632 2.4271621178 |
| H -0.78189 -0.56367 2.53898 | H -0.677782637 -0.7661389828 2.4763541744 |
| H 0.8719 -0.32682 2.53296 | H 0.9030706763 -0.290299356 2.4806741543 |
| H -0.15585 0.94501 -2.34212 | H -0.2973972656 0.8194286255 -2.4297879393 |
| H -0.78178 -0.56441 -2.53885 | H -0.6799135326 -0.770812476 -2.4750028236 |
| H 0.87192 -0.32703 -2.53288 | H 0.9015367386 -0.297115522 -2.481502379 |
| cis-$[Ga(NH_3)_2Cl_4]^-$ gas phase | cis-$[Ga(NH_3)_2Cl_4]^-$ solution (liq. $NH_3$) |
| E = -3879.2343634 | E = -3879.332284 |
| Ga -4.3788710369 0.1943711294 -0.1390206779 | Ga -4.383868 0.1356868275 -0.1417274996 |
| Cl -2.0618266189 -0.5101235253 0.033994653 | Cl -2.0266058919 -0.3322162656 0.0883880049 |
| Cl -6.7808587168 -0.0814363677 -0.3585401725 | Cl -6.7845136691 0.1041789015 -0.395634 |
| Cl -4.4044017405 1.5786303245 1.6822606675 | Cl -4.4304724457 1.6297679052 1.6562977604 |
| Cl -4.100415951 1.7192167424 -1.8216735745 | Cl -4.0661303607 1.7623804785 -1.7910431651 |
| N -4.6476450842 -1.452527885 1.2942773979 | N -4.6591239507 -1.4562993393 1.2446971501 |
| N -4.3855358162 -1.3314486099 -1.7233987844 | N -4.375272056 -1.341803398 -1.6744948159 |
| H -3.5969291397 -1.9581096438 -1.6348083485 | H -3.5290844229 -1.8996602186 -1.6483915712 |
| H -4.2635507012 -0.7361823381 -2.5348788587 | H -4.3978712072 -0.8482427168 -2.5601498791 |
| H -5.2774106521 -1.8050884984 -1.775115224 | H -5.1829119602 -1.9528634087 -1.6386908723 |
| H -4.6723457608 -0.924261009 2.1591787737 | H -4.5593335268 -1.0598722278 2.1728046714 |
| H -3.8507517087 -2.0749535223 1.2939906251 | H -3.96832065 -2.1909868389 1.1447792715 |
| H -5.5313560729 -1.9233447967 1.1534445233 | H -5.5883908588 -1.855327699 1.172875945 |
| trans-$[Ga(NH_3)_2Cl_4]^-$ gas phase | trans-$[Ga(NH_3)_2Cl_4]^-$ solution (liq. $NH_3$)* |
| E = -3879.2397067 | E = -3879.3345362 |
| Ga -2.3161265999 0.0319154304 -0.0223942042 | Ga -2.3235231896 0.0250235002 -0.0163691296 |
| Cl 0.0513810438 0.0249789069 0.0293004538 | Cl 0.0697118894 0.0336531745 0.0203706556 |
| Cl -4.702537335 0.026306446 -0.0630991795 | Cl -4.7252721809 0.0023150362 -0.0410873076 |
| Cl -2.3542530245 -1.7491762877 1.5678956943 | Cl -2.3367225966 -1.7639836346 1.5886204056 |
| Cl -2.2935935079 1.797380266 -1.5989646579 | Cl -2.3209195939 1.8057187454 -1.6109672111 |
| N -2.2909376436 -1.3693551126 -1.5827474131 | N -2.2916442824 -1.3521527103 -1.569263874 |
| N -2.3505372628 1.4245499018 1.5455975648 | N -2.3493254193 1.4103377144 1.5289620328 |
| H -2.1342233903 0.9208776121 2.396745829 | H -2.0725659379 0.9672128913 2.3979706225 |
| H -1.6593279526 2.1371386226 1.3522084042 | H -1.7127996681 2.1744064686 1.3336427273 |
| H -3.2841895357 1.81427705 1.5801153676 | H -3.2847433975 1.7856444369 1.6411142324 |
| H -2.0757394031 -2.2693459615 -1.1720542645 | H -2.2097066023 -2.2923029474 -1.1975992419 |
| H -1.5836386402 -1.0887771563 -2.2490526784 | H -1.5030640715 -1.1744149108 -2.180888368 |
| H -3.2131517482 -1.3703657177 -2.0001819161 | H -3.1462999493 -1.2910537643 -2.111136544 |
| $[Ga(NH_3)Cl_5]^{2-}$ gas phase | $[Ga(NH_3)Cl_5]^{2-}$ solution (liq. $NH_3$) |
| E = -4282.8474725 | E = -4283.1251487 |
| Ga -4.4327863096 0.1404200505 -0.2044779303 | Ga -4.4051932644 0.0942025564 -0.1764063874 |
| N -4.5485652303 -1.4125851902 1.2471804849 | Cl -2.0135383598 -0.1920524639 0.1213437334 |
| Cl -2.0249569496 -0.1055411188 0.1640141778 | Cl -6.8280145958 0.1577443315 -0.2231970665 |

| | |
|---|---|
| Cl -6.897400733 -0.0552186097 -0.138575935 | Cl -4.4224294591 1.6827845361 1.6628382391 |
| Cl -4.5493743466 1.5842135474 1.808383627 | Cl -4.419354964 -1.7184697046 -1.7773800033 |
| Cl -4.3633423928 -1.7340794321 -1.7720677617 | Cl -4.1717776867 1.736108461 -1.7974330779 |
| Cl -4.3320699534 1.819924715 -1.7335196233 | N -4.6229584301 -1.4001062508 1.2846616569 |
| H -4.4069173646 -0.9376810106 2.1293776973 | H -4.6992632725 -0.9663305104 2.198122917 |
| H -3.8188440118 -2.0723251962 1.0216522311 | H -3.8154862549 -2.0120827689 1.2745097605 |
| H -5.4826367083 -1.7870687552 1.1476660323 | H -5.4588767126 -1.9417401864 1.1025752282 |

| $[GaCl_5]^{2-}$ gas phase<br>E = -4226.2759 | $[GaCl_5]^{2-}$ solution (liq. $NH_3$)<br>E = -4226.5431737 |
|---|---|
| Ga 0. 0.0732 -0.1267861466 | Ga 0.0000000002 0.0733363333 -0.1270222829 |
| Cl 0. -1.0681165401 1.8500332052 | Cl -0.0000000003 -1.052847316 1.8235861194 |
| Cl 0. -1.0681175073 -2.1036049399 | Cl 0.0000000005 -1.0394416998 -2.0867060973 |
| Cl 0. 2.3558340474 -0.126786705 | Cl 0.0000000005 2.3268613086 -0.1431712485 |
| Cl 2.4390895267 0.0732 -0.1267861466 | Cl 2.4408562904 0.0656456871 -0.1137016854 |
| Cl -2.4390895267 0.0732 -0.1267861466 | Cl -2.4408562899 0.0656456876 -0.1137016863 |

| $[GaCl_6]^{3-}$ gas phase<br>E = -4686.3244917 | $[GaCl_6]^{3-}$ solution (liq. $NH_3$)*<br>E = -4686.90428803 |
|---|---|
| Ga -4.4434604634 0.0884323782 -0.1384746267 | Ga -0.000129 -0.000098 -0.000075 |
| Cl -1.9818597671 0.0540774121 -0.0034811739 | Cl 0.235439 -1.118605 -2.132994 |
| Cl -4.5634762794 -1.7215724621 1.5317512386 | Cl -0.236869 1.116873 2.133524 |
| Cl -6.9067423329 0.1266633438 -0.2764531381 | Cl 2.239085 0.891422 -0.219995 |
| Cl -4.5192287276 1.7614540491 1.669159919 | Cl -2.239623 -0.891256 0.218321 |
| Cl -4.367557975 -1.5873155788 -1.9490519582 | Cl 0.888184 -1.951670 1.121786 |
| Cl -4.3297654546 1.9006658577 -1.8084162608 | Cl -0.885981 1.953414 -1.120505 |

| $[Ga(NH_3)_5(NH_2)]^{2+}$ gas phase<br>E = -2263.3035498 | $[Ga(NH_3)_5(NH_2)]^{2+}$ solution (liq. $NH_3$)*<br>E = -2263.580047 |
|---|---|
| Ga 0.0332591738 0.0606026878 -0.0235632162 | Ga -0.001834 0.001564 -0.103316 |
| N -1.5503847125 -0.8092359355 1.1545708456 | N 0.183642 -0.137445 2.025680 |
| N 1.3901790846 0.8164099004 -1.0502038155 | N -0.188022 0.111930 -1.993592 |
| N -1.5146814855 0.7973772634 -1.3998407214 | N -1.993331 0.763390 0.219089 |
| N 0.1006933462 -1.7787307984 -1.1978125585 | N -0.830036 -1.985757 -0.063375 |
| N -0.2028703476 1.9072584188 1.117537258 | N 0.696562 2.034262 0.030771 |
| N 1.3611719517 -0.8092010975 1.5352800153 | N 2.045797 -0.756118 -0.090577 |
| H -1.4664119474 -1.8188415127 1.2481384067 | H 0.442205 -1.071193 2.328799 |
| H -2.4790948907 -0.6435318288 0.773466553 | H -0.675354 0.091129 2.516315 |
| H -1.5757743352 -0.454713988 2.1078680253 | H 0.895785 0.481863 2.400331 |
| H -2.17698885 1.498815937 -1.0778770722 | H -2.076123 1.628784 0.741924 |
| H -2.06610559 0.131143305 -1.9349931854 | H -2.665762 0.121136 0.625086 |
| H -0.8997511334 1.2650541216 -2.066588208 | H -2.300869 0.952851 -0.730846 |
| H 0.895414555 -2.3677114411 -0.9594599298 | H -0.145477 -2.710742 -0.252909 |
| H -0.7033707939 -2.4003743085 -1.233016765 | H -1.311278 -2.263394 0.786245 |
| H 0.2699772457 -1.4896642054 -2.1597337224 | H -1.513030 -2.044303 -0.813274 |
| H 0.5341884543 2.0391316097 1.8064868482 | H 1.681920 2.129951 -0.193605 |
| H -1.0738140988 2.1087612881 1.6023711089 | H 0.556856 2.512579 0.914904 |
| H -0.0747922839 2.6665777253 0.4508171766 | H 0.198872 2.577573 -0.668502 |
| H 1.2853435371 -0.4575373795 2.4868801937 | H 2.728175 -0.181940 0.394970 |
| H 1.3979614351 -1.8214148892 1.6303182349 | H 2.170266 -1.695614 0.273103 |
| H 2.280848257 -0.5243380924 1.2026298835 | H 2.329718 -0.784834 -1.064970 |
| H 1.8832024874 1.6541975243 -0.7822978079 | H 0.434116 0.760132 -2.459294 |
| H 2.000588941 0.2748166958 -1.6425055475 | H -0.095463 -0.764303 -2.491452 |

| trans-$[Ga(NH_3)_4(NH_2)_2]^{+}$ gas phase<br>E = -2263.0240923 | trans-$[Ga(NH_3)_4(NH_2)_2]^{+}$ solution (liq. $NH_3$)<br>E = -2263.102331 |
|---|---|
| Ga -0.0004076965 0.0043977544 0.0154216953 | Ga -0.01001675 -0.0045054488 -0.0049435231 |
| N -1.4358454539 -0.7311938992 0.9760264022 | N -1.4569416104 -0.7500323094 1.0033537459 |
| N 1.414513981 0.7211935578 -0.9887976013 | N 1.4417025116 0.7453970325 -1.0030767025 |
| N -1.5133640591 0.7341726758 -1.513216939 | N -1.4547268916 0.7275176648 -1.5247159432 |

| | |
|---|---|
| N 0.2052906886 -1.8938656015 -1.2155532662 | N 0.1560076371 -1.8793123628 -1.1840790522 |
| N -0.351902645 1.9478800021 1.2514833299 | N -0.3383645647 1.9099940199 1.1970220198 |
| N 1.6283024739 -0.7986627427 1.4754977818 | N 1.5719053775 -0.798882587 1.4386872307 |
| H -1.5431923943 -1.7348398266 0.9654944015 | H -1.4304464397 -1.7623621351 1.0340145599 |
| H -1.6035008641 -0.4143035596 1.9193157987 | H -1.4633742049 -0.4474691261 1.9700916097 |
| H -1.6465399049 1.7372833602 -1.5728994036 | H -1.5872067669 1.7323513997 -1.5118097678 |
| H -2.3567999056 0.3303819302 -1.115712059 | H -2.3375067881 0.3000157448 -1.2685490259 |
| H -1.4116221222 0.4011333502 -2.465230272 | H -1.242802535 0.4712520391 -2.4820595876 |
| H 0.5421960485 -2.7147740537 -0.7255204062 | H 0.539788883 -2.6583189894 -0.661329599 |
| H -0.6014716061 -2.1912856042 -1.7524651571 | H -0.7072250903 -2.2040552004 -1.604149953 |
| H 0.9321215353 -1.5944817889 -1.8593771191 | H 0.8089659929 -1.6594054233 -1.9276916687 |
| H -0.017796091 1.907052767 2.2080406139 | H 0.0513461241 1.8701134473 2.1319231205 |
| H -1.3375497396 2.1825096205 1.3039413476 | H -1.3247237055 2.1206356681 1.3008760286 |
| H 0.1297878364 2.7189278777 0.8029302043 | H 0.0974393402 2.6949406273 0.7274943866 |
| H 2.0354529932 -0.0961256386 2.0829267892 | H 1.927123113 -0.0937614084 2.0744000144 |
| H 1.2181330361 -1.5057926686 2.0750547894 | H 1.1827516928 -1.5416329864 2.0073672357 |
| H 2.400994968 -1.2234667372 0.9737158862 | H 2.372734619 -1.1755486172 0.9438644536 |
| H 2.2560327715 1.0058289225 -0.5101129569 | H 2.1852040161 1.1012383804 -0.4142198925 |
| H 1.1938911498 1.4316173023 -1.6711168596 | H 1.1550910396 1.5254175704 -1.5826226887 |

| cis-$[Ga(NH_3)_4(NH_2)_2]^+$ gas phase<br>E = -2263.0167529 | cis-$[Ga(NH_3)_4(NH_2)_2]^+$ solution (liq. $NH_3$)<br>E = -2263.0992846 |
|---|---|
| Ga -1.5550910437 -0.928873347 1.1623031453 | Ga 0.0477097613 0.204296 -0.0249852312 |
| N 0.0533285896 0.1842345385 -0.0051099127 | N -1.5438915915 -0.8727280364 1.1223907013 |
| N -2.9360173241 -0.0154566971 -0.3491270877 | N 1.3908545241 -0.7894398122 1.4171117729 |
| N -2.8694959175 -1.4672035294 2.4276569411 | N 1.4137607847 0.8296309112 -1.2367313668 |
| N 0.0063238797 -1.483511548 2.672223135 | N -1.541742742 0.7224983372 -1.468306231 |
| N -1.2049454434 -2.310951705 -0.0969963588 | N 0.1038900657 -1.7092590959 -1.1808742847 |
| N -1.6194803014 1.0313099048 2.3192791054 | N -0.2467610304 1.6734641753 1.1916234903 |
| H -0.0096999951 -0.316088193 -0.8877013245 | H -1.4646462849 -1.8807597587 1.1914062945 |
| H 0.9951938445 0.047045049 0.3445249833 | H -2.4756507723 -0.6750631752 0.7749051908 |
| H -0.0479927222 1.1734338956 -0.2012201347 | H -1.4988907764 -0.5039397081 2.0665405529 |
| H 0.7201407847 -0.882008914 3.0672527032 | H -2.1419677005 1.4520610752 -1.1013681008 |
| H 0.4581293171 -2.3442023044 2.382400675 | H -2.1389958544 0.0116181584 -1.8747920006 |
| H -0.650762424 -1.7243100748 3.4119845388 | H -0.9909987828 1.1285844885 -2.2179514689 |
| H -0.2635729445 -2.6567266052 -0.2220190575 | H 0.811692953 -2.3508665988 -0.841196034 |
| H -1.8022185847 -3.1238317515 -0.024160151 | H -0.7601549166 -2.2347063605 -1.2474670292 |
| H -2.2929447708 1.7029399755 1.9677993832 | H 0.3711471174 -1.4518037996 -2.1251822737 |
| H -0.7649749629 1.5418062176 2.510194335 | H -1.2179997369 1.9063382097 1.3612049916 |
| H -1.9807122669 0.6868431591 3.2047903838 | H 0.1883601774 2.5309340518 0.8716927461 |
| H -2.8803600054 0.9140389586 -0.7495387329 | H 1.3952009913 -0.110284306 2.1713818451 |
| H -2.7387654834 -0.6904456403 -1.0856497693 | H 1.1606601021 -1.692219069 1.8162602923 |
| H -3.8955621861 -0.1650023839 -0.0561791359 | H 2.3353922597 -0.8482590253 1.0543794168 |
| H -3.7086805232 -0.9180278483 2.5523247673 | H 1.8523832389 1.684286472 -0.9141336699 |
| H -3.1649925163 -2.4320661568 2.3603395685 | H 2.1723512132 0.1797248665 -1.4053976035 |

| $[Ga_2(NH_3)_6(\mu\text{-}NH_2)_2]^{4+}$ gas phase<br>E = -4413.1103173 | $[Ga_2(NH_3)_6(\mu\text{-}NH_2)_2]^{4+}$ solution (liq. $NH_3$)<br>E = -4414.0120274 |
|---|---|
| Ga -0.1214530267 0.0844047708 -0.0543012071 | Ga -0.1163167265 0.1045331756 -0.0981992857 |
| Ga -0.0837851956 1.8164150127 -2.7417385509 | Ga -0.0887189274 1.7861908306 -2.7042204059 |
| N -1.7119397129 -0.7649760024 1.153219214 | N -1.6919179176 -0.6812031371 1.1346576514 |
| N 1.4338452527 -0.6845352948 1.2491376816 | N 1.4279388842 -0.6838204697 1.1797557345 |
| N 1.2248752318 0.9907870304 -1.3533959658 | N 1.23136335 0.9663996432 -1.370953914 |
| N -1.4301165388 0.9100597654 -1.442635652 | N -1.4346004931 0.8864354893 -1.4560212799 |
| N -0.0313429092 -1.8901279508 -0.9926375293 | N -0.0916341752 -1.8634232816 -0.9515725728 |
| N -0.2181701226 1.7557552275 1.3544643751 | N -0.1418610289 1.7284280687 1.3103769061 |
| N -1.6383782342 2.570036145 -4.0549734645 | N -1.6295089728 2.5159284154 -4.0201072642 |
| N -0.1768641719 3.7909222107 -1.8036590497 | N -0.2410301756 3.7348781887 -1.809516881 |
| N 0.0158726231 0.1452401397 -4.1504879809 | N 0.0647612455 0.201225316 -4.141143972 |
| N 1.5060239056 2.6809801641 -3.9393633885 | N 1.4801666919 2.6959762534 -3.85809629 |

| | |
|---|---|
| H -1.4298972149 -1.220876979 2.0222165162 | H -1.3930525785 -1.1277178851 1.9969449736 |
| H -2.2644311473 -1.4715243073 0.6670024529 | H -2.2541550505 -1.3719543128 0.6462457171 |
| H -2.4038314063 -0.0740003945 1.4443508257 | H -2.3437578638 0.0485194304 1.4087571201 |
| H -2.1026978068 1.5573279346 -1.0368460766 | H -2.117051074 1.5219823043 -1.0549626991 |
| H -2.0335054731 0.2240463909 -1.8912939424 | H -2.0078885079 0.1863575682 -1.9162423035 |
| H 0.7985951023 -2.0780346834 -1.5533301513 | H 0.7238997893 -2.0818906032 -1.516395447 |
| H -0.0212697715 -2.6301324075 -0.2887880397 | H -0.1023535401 -2.5611470487 -0.2131745909 |
| H -0.8202259486 -2.1338639596 -1.5900279929 | H -0.8973538857 -2.0724464053 -1.5345978073 |
| H 0.5791791741 2.3903967413 1.3453431927 | H 0.687978438 2.3136121945 1.2911026789 |
| H -0.2515626299 1.4207095438 2.318695755 | H -0.1738067304 1.3476504469 2.2519117204 |
| H -1.0392069642 2.3544908207 1.2776698309 | H -0.9350811693 2.3600698079 1.2545490526 |
| H 1.1289081081 -1.2842460828 2.0171940435 | H 1.1116968566 -1.2855782495 1.9349272104 |
| H 2.1288160337 -1.2435552918 0.7537271748 | H 2.0949770283 -1.2339854475 0.6460378049 |
| H 1.9865384082 0.0430288954 1.7030584725 | H 1.9786966358 0.047190249 1.6204769021 |
| H 1.8281948595 1.6750644254 -0.9020012569 | H 1.8473539017 1.6402388383 -0.927159016 |
| H 1.8975314879 0.3453192508 -1.7619202678 | H 1.873858448 0.3017950503 -1.7905923454 |
| H 0.6006311287 4.0277878173 -1.1889405019 | H 0.5087137475 3.999153198 -1.1776001446 |
| H -0.1645099509 4.5315135856 -2.5068568238 | H -0.2271112167 4.4370728701 -2.5437935224 |
| H -1.3269933738 3.0438934125 -4.9041183167 | H -1.3055115493 2.9782947642 -4.8648872539 |
| H -2.2659543441 1.8388569453 -4.3902996076 | H -2.2306508922 1.7578713881 -4.3306504317 |
| H 1.2176160683 3.2624023606 -4.7276004843 | H 1.1738466829 3.2750005517 -4.634749851 |
| H 2.1307371854 3.2803750932 -3.3995694258 | H 2.0659693131 3.29116506 -3.2792444464 |
| H 0.0270088644 0.479718214 -5.1154314297 | H 0.0906005738 0.5866363115 -5.080787326 |
| H 0.8478448695 -0.4398787089 -4.0866356196 | H 0.909390141 -0.3569677131 -4.052950458 |
| H -1.0173938713 3.9851797048 -1.2610783183 | H -1.107277071 3.8985362272 -1.3053979038 |
| H -2.2641084516 3.2407638904 -3.6083775136 | H -2.2503928144 3.1774085088 -3.5630026204 |
| H 2.1312918485 1.9892152732 -4.3533232735 | H 2.1113876563 2.0081557535 -4.2584109763 |
| H -0.7706189006 -0.5023716639 -4.1278186612 | H -0.706314034 -0.460004409 -4.1426833404 |

* lowest energy configuration of non-converged optimization run

**Table S2:** Atomic coordinates (in Å) and energies (in a.u.) for ion groups $\{[Ga(NH_3)_6]X_n\}^{(3-n)+}$ obtained after energy optimization at the PCM-ωB97xD/def2-tzvpp level (in 'solution').

| $\{[Ga(NH_3)_6]Cl\}^{2+}$ solution (liq. $NH_3$) | $\{[Ga(NH_3)_6]Cl_2\}^{+}$ solution (liq. $NH_3$)<br>E = -3184.886745 |
|---|---|
| Ga -0.618019 0.001789 0.003068 | Ga -0.0001826857 0.0004804827 -0.0000843525 |
| N 0.616681 1.430427 0.896599 | N -1.2638512442 -1.4999534245 0.7629922083 |
| N -1.887432 -1.393588 -0.985315 | N -1.264341262 0.0846627711 -1.6787841648 |
| N -1.883470 -0.153497 1.701709 | N 1.2638815818 1.5009303627 -0.7636048044 |
| N 0.609880 -1.491506 0.795557 | N 1.2639249424 -0.0827656125 1.6782486749 |
| N -1.863478 1.561124 -0.725010 | N -1.266859635 1.4116403686 0.9131937192 |
| N 0.608046 0.045821 -1.686375 | N 1.2662507369 -1.4105104093 -0.9140756859 |
| H 0.497408 1.546352 1.897932 | H -1.1540276394 -1.6779167002 1.7563672898 |
| H 0.534451 2.359680 0.495555 | H -1.1688384666 -2.3978106512 0.2990376344 |
| H 1.596460 1.137494 0.749166 | H -2.2422478966 -1.2050154381 0.6257875873 |
| H -2.776713 0.319455 1.595178 | H 2.2432104758 -0.0606446021 1.3548922301 |
| H -1.473379 0.231030 2.547464 | H 1.1651955796 -0.9278810088 2.2316429872 |
| H -2.106265 -1.120772 1.920878 | H 1.1562845621 0.6944741241 2.3219237912 |
| H 1.590996 -1.205272 0.640976 | H -2.245399375 1.140130303 0.7344275772 |
| H 0.507088 -1.634977 1.795859 | H -1.1658500124 1.4718007039 1.9212298257 |
| H 0.510144 -2.406205 0.366840 | H -1.1614663703 2.3565747006 0.5580996032 |
| H -1.441987 2.099060 -1.477064 | H 1.1653978214 -1.4696019774 -1.9221662904 |
| H -2.095328 2.237286 -0.002710 | H 1.1604080434 -2.3557102218 -0.5598293175 |
| H -2.752038 1.230156 -1.089214 | H 2.2449386367 -1.1394777396 -0.7349011932 |
| H 0.503975 0.865121 -2.276379 | H -1.1555390686 -0.6909836649 -2.3241485799 |
| H 1.590031 0.038607 -1.366199 | H -2.2435870141 0.060850895 -1.3554030506 |
| H 0.500124 -0.756118 -2.299927 | H -1.1666574002 0.9312222381 -2.2301859578 |
| H -2.062469 -1.132636 -1.951965 | H 1.1546038458 1.6783289724 -1.7571509635 |
| H -2.802607 -1.496287 -0.555776 | H 2.2424189512 1.2060104232 -0.6259832205 |
| H -1.499285 -2.331931 -1.022867 | H 1.1686535468 2.399095075 -0.3002612305 |
| Cl 3.349200 -0.001586 -0.002529 | Cl -4.0320035568 -0.004526048 -0.0008702834 |
| | Cl 4.0322339029 0.004245078 0.0002119666 |

| $\{[Ga(NH_3)_6]Cl_3\}$ solution (liq. $NH_3$)<br>E = -3645.2912033 | -4105.6907463 |
|---|---|
| Ga -0.6385999495 0.030920046 0.0032352178 | Ga 0.0034494085 -0.0018843434 -0.0499108294 |
| N 0.6420696676 1.4446344671 0.8972138989 | N 0.4683270587 1.5256527953 -1.4122421743 |
| N 0.5776333694 0.1849019499 -1.6995739083 | N 1.6256632947 0.6893219224 1.0754931561 |
| N -1.9462338496 -1.3903749229 -0.8109313959 | N -0.2217065284 -1.5979454682 1.2839714339 |
| N -1.7794804077 -0.0866102369 1.7674400677 | N -1.7049671419 -0.4463624132 -1.1902112825 |
| N 0.5735959097 -1.5418570202 0.646445646 | N -1.2747643923 1.145660598 1.1453450312 |
| N -1.8679497374 1.4939256566 -0.8978371905 | N 1.1142773344 -1.3407403618 -1.2246311454 |
| H 0.5817255243 1.4961223472 1.9085944453 | H 0.1207384442 2.434786442 -1.1199769017 |
| H 0.5209606524 2.3943706613 0.5614791219 | H 0.06563103 1.3822454507 -2.3324125983 |
| H 1.6114597968 1.1626514602 0.6776913752 | H 1.4902679172 1.5557891672 -1.5205029168 |
| H -2.6681202321 0.4018152851 1.7417283854 | H -1.4957256931 -0.8545971033 -2.0952253085 |
| H -1.3011490426 0.2479239069 2.5969608266 | H -2.2178595136 0.4305944615 -1.3442758656 |
| H -1.9824967459 -1.0857713395 1.9348496966 | H -2.3163080381 -1.1213912174 -0.7405927928 |
| H 1.5109930135 -1.2289030768 0.9106707863 | H -0.8484455458 1.3555031496 2.0505578866 |
| H 0.138837602 -2.0862826814 1.3949339169 | H -1.5789363488 1.9937129899 0.6588038584 |
| H 0.7170143741 -2.1997443861 -0.1141894348 | H -2.1273600466 0.6376896115 1.3595485464 |
| H -1.4639382468 2.4230245124 -0.9528464331 | H 2.048666006 -1.501523711 -0.8612046094 |
| H -2.7868832755 1.6147530215 -0.4846607988 | H 1.2614776429 -1.0207007923 -2.175871956 |
| H -2.012373651 1.1717713763 -1.8667055928 | H 0.6025355428 -2.2318178024 -1.2622146824 |
| H 0.7321563044 1.1490555193 -1.9787302974 | H 2.5161562774 0.469204665 0.6212748274 |
| H 1.5090403201 -0.193665774 -1.5138418911 | H 1.6073174087 1.7015493597 1.1525456831 |
| H 0.1502764559 -0.2632594225 -2.5135723651 | H 1.5876073579 0.340601669 2.0360299768 |
| H -1.8071410215 -1.4994772497 -1.8179132193 | H 0.6626948565 -2.0729662745 1.4346470518 |
| H -2.9196230174 -1.1208417949 -0.7098786616 | H -0.8508589035 -2.3116567923 0.9065194008 |

H -1.8589070746 -2.2859741579 -0.324355526
Cl 3.387932308 -0.0236942923 0.0643695205
Cl -1.8630947586 -3.3040897643 1.9010964848
Cl -1.878941288 -0.1253390903 -3.7880936751

H -0.5189003163 -1.2747523591 2.2072634684
Cl 0.1418108627 0.2545671447 3.9780488971
Cl -2.5950748584 2.7606291628 -1.3290213973
Cl 3.6867419553 0.7593960841 -1.4157858787
Cl -1.1740990713 -3.7101220345 -0.9836228792

$\{[Ga(NH_3)_6]Cl_5\}^{2-}$ solution (liq. $NH_3$)
E = -4566.0962326

Ga -0.0049433594 -0.0045711828 0.0071901662
N -1.0594612657 -1.4047982312 -1.1269370042
N -0.6843837261 1.6404837448 -1.1205159356
N -1.7760537616 0.2328868446 1.0875206111
N 0.6924368293 -1.6392113052 1.1036832629
N 1.7716325231 -0.2223333936 -1.1042732475
N 1.0350203063 1.37674451 1.2043779492
H -1.7942523937 -0.9842368916 -1.6993262359
H -1.586912202 1.4097351931 -1.5562837342
H -0.4515743873 -2.0023878941 -1.6907137547
H -0.0691503189 1.9971606393 -1.842163626
H -1.5293999482 -2.0188103325 -0.4547110461
H -1.7940377591 1.0887497194 1.6446942768
H -2.5361315082 0.3157794198 0.4064292761
H -1.9909786814 -0.5868873331 1.6600033138
H 0.9827560189 -2.3530840592 0.4294684979
H 1.5168415773 -1.4222024975 1.6663051374
H -0.0436839788 -2.0636242779 1.6728501622
H 1.7963061028 -1.1566130637 -1.5334196281
H 2.5511532783 -0.1941763565 -0.4390022693
H 1.955047599 0.4613794433 -1.8291065661
H 0.4033667565 1.9472821886 1.7713873074
H 1.7512392768 0.9257730916 1.7785672844
H 1.5380756778 2.0366624417 0.6194326147
H -0.8734147645 2.4027274631 -0.4615342504
Cl 3.6954278626 -0.3964741295 1.731076319
Cl -2.2378720706 -2.9339730652 1.7641254735
Cl -1.394800502 3.4626591548 1.6989882613
Cl 1.4885517311 -3.3746004689 -1.7964855474
Cl -3.6377359123 0.5128776285 -1.8301540685

$\{[Ga(NH_3)_6]Cl_6\}^{3-}$ solution (liq. $NH_3$)
E = -5026.4882041

Ga -0.0010030341 0.0010980519 -0.00178244
N -1.7038647068 -0.5143101995 -1.1091374739
N 0.4048762875 1.7283394962 -1.1163143014
N -1.2928672136 1.2189671557 1.1092569942
N -0.4043116342 -1.7269155935 1.1112757118
N 1.2896331363 -1.2152764257 -1.1155587004
N 1.7014234447 0.5144519085 1.1060172396
H -2.0553863359 0.2615392531 -1.6746021875
H -0.3942058218 2.0320455912 -1.6764238311
H -1.5680937097 -1.355078777 -1.6743739295
H 1.2481331542 1.6448117255 -1.6881216447
H -2.4361312054 -0.7343958405 -0.4296061214
H -0.7965272104 1.9097670227 1.6760023715
H -1.8507583962 1.745727679 0.4320617293
H -1.9532239965 0.6816361636 1.6746987535
H -0.5899754331 -2.4702225799 0.4326007614
H 0.3969675098 -2.032087421 1.667482359
H -1.2450333065 -1.6432250261 1.6866487258
H 0.7929646072 -1.9067138578 -1.6809489026
H 1.8492346464 -1.7415489231 -0.4393093902
H 1.9485174489 -0.6771293213 -1.6815425631
H 1.5648387527 1.3529426777 1.6742348057
H 2.0535086652 -0.2628973604 1.6689007139
H 2.4334449323 0.7371170228 0.4269021933
H 0.5870665558 2.4729497074 -0.4384617947
Cl 2.7089086603 -2.5345652875 1.8041166944
Cl -3.5383988139 -1.0617424227 1.8510379116
Cl 0.8425713963 3.6077696635 1.8245900419
Cl -0.8447058179 -3.6094463788 -1.8210965531
Cl -2.7081046139 2.531893738 -1.815720466
Cl 3.5478890521 1.061062558 -1.8319847073

$\{[Ga(NH_3)_6]Br\}^{2+}$ solution (liq. $NH_3$)*
E = -4838.472177

Ga 1.158260 0.002839 0.004407
N -0.066371 0.685630 -1.549366
N 2.387220 -0.639994 1.609632
N 2.448705 -1.048110 -1.319053
N -0.066669 -1.692776 0.119408
N 2.390471 1.714309 -0.253128
N -0.078002 0.974666 1.380306
H 0.038382 0.185167 -2.426759
H 0.053766 1.669526 -1.771517
H -1.052308 0.570894 -1.272822
H 3.408056 -0.712109 -1.323260
H 2.147275 -1.017630 -2.288738
H 2.505797 -2.035238 -1.082995
H -1.052147 -1.391653 0.137127
H 0.020028 -2.310417 -0.683065
H 0.067672 -2.277853 0.938204
H 1.929536 2.586109 -0.010375
H 2.685876 1.825679 -1.219552

$\{[Ga(NH_3)_6]Br_2\}^{+}$ solution (liq. $NH_3$)*
E = -7412.904808

Ga 0.000094 -0.000336 0.000648
N 1.262937 -0.089500 1.682440
N 1.260967 1.507117 -0.757869
N -1.262064 0.090062 -1.681662
N -1.261788 -1.505149 0.762271
N 1.261884 -1.414610 -0.915790
N -1.262167 1.415012 0.915846
H 1.155442 -0.929523 2.242809
H 1.158151 0.692945 2.320297
H 2.245234 -0.076416 1.374327
H -2.243798 -1.232670 0.620577
H -1.158941 -1.675449 1.758219
H -1.151740 -2.405800 0.307085
H 2.244218 -1.153727 -0.754502
H 1.158892 -2.356902 -0.551165
H 1.151171 -1.484714 -1.923077
H -1.161276 2.356693 0.548355
H -1.149790 1.487775 1.922740

| | |
|---|---|
| H 3.246751 1.694432 0.294541 | H -2.244339 1.151513 0.756966 |
| H 0.036016 1.982814 1.419518 | H 1.154189 2.404334 -0.294941 |
| H -1.062192 0.806749 1.123781 | H 2.243008 1.231604 -0.623591 |
| H 0.022721 0.645320 2.335550 | H 1.153267 1.685257 -1.751842 |
| H 2.640871 0.125753 2.228411 | H -1.157896 0.933554 -2.237566 |
| H 3.264203 -1.065925 1.324309 | H -2.244458 0.071581 -1.374322 |
| H 1.934000 -1.331320 2.201341 | H -1.153721 -0.688500 -2.323712 |
| Br -3.024224 0.000161 0.000717 | Br 4.245711 -0.000379 -0.001763 |
| | Br -4.245680 -0.000240 -0.000334 |
| {$[Ga(NH_3)_6]Br_3$} solution (liq. $NH_3$)*<br>E = -9987.32203091 | {$[Ga(NH_3)_6]I_3$} solution (liq. $NH_3$)<br>E = -3158.0295311 |
| Ga 0.022204 -0.005517 0.806528 | Ga -0.6208593545 0.054034283 -0.0345034137 |
| N -1.069054 -1.408625 1.936321 | N 0.6984796363 1.4186071093 0.8862338096 |
| N 0.780586 -1.626840 -0.288224 | N -1.7973054319 1.6001495909 -0.8528489787 |
| N 1.062333 1.457136 -0.275909 | N 0.6354721546 0.1289398432 -1.7358009936 |
| N -0.813047 1.564939 1.939173 | N -1.9507072622 -1.3353262651 -0.8734816394 |
| N -1.533302 0.139903 -0.583619 | N -1.8460545662 0.000577418 1.6593127938 |
| N 1.749023 -0.166712 2.015691 | N 0.564502567 -1.5370701147 0.6423851959 |
| H -1.857357 -1.017421 2.441568 | H 0.6282673132 1.4227195614 1.8991499312 |
| H -0.522225 -1.916117 2.624179 | H 0.5868977949 2.3836721468 0.5915160613 |
| H -1.464763 -2.108786 1.292461 | H 1.6726648267 1.1680690011 0.6767818704 |
| H -0.203087 1.953040 2.651434 | H -2.8117009888 0.2445468081 1.4166388529 |
| H -1.675857 1.322369 2.415222 | H -1.5786397491 0.6054354761 2.4285984353 |
| H -1.053461 2.334515 1.298267 | H -1.8984304144 -0.9531292513 2.034349315 |
| H -2.216451 -0.614487 -0.497899 | H 1.5171763962 -1.255831209 0.8816638003 |
| H -2.007154 1.045862 -0.548128 | H 0.1582562806 -2.027679539 1.4409962346 |
| H -1.172432 0.062423 -1.530417 | H 0.6649651895 -2.2472619011 -0.0775349417 |
| H 1.780998 -0.987729 2.611440 | H -1.5675801441 2.5339977973 -0.5286531157 |
| H 1.934407 0.623773 2.624993 | H -2.7885493198 1.4536905114 -0.6243646871 |
| H 2.551384 -0.238187 1.375317 | H -1.7553944006 1.6324114464 -1.8672449799 |
| H 1.199397 -2.335155 0.307387 | H 0.6226619197 1.0269379214 -2.2103754408 |
| H 0.036373 -2.106587 -0.801088 | H 1.6119879604 -0.0228852936 -1.4579689006 |
| H 1.525057 -1.350571 -0.930335 | H 0.4263615284 -0.5682285394 -2.4436912947 |
| H 1.794558 1.057287 -0.867085 | H -1.748335078 -1.6438814911 -1.818763481 |
| H 1.544497 2.108880 0.335510 | H -2.9065194825 -0.9659305201 -0.8812772425 |
| H 0.431399 2.031448 -0.838497 | H -1.9914141348 -2.1735023989 -0.285364138 |
| Br -2.303162 -3.219718 -0.677233 | I 3.9241750315 -0.0612780344 -0.0420960592 |
| Br -1.697356 3.578534 -0.672623 | I -2.0382552392 -3.648719104 2.1090665802 |
| Br 3.927679 -0.342020 -0.712163 | I -5.1441930536 0.430552792 -0.0122835694 |
| {$[Ga(NH_3)_5(NH_2)]Cl_2$} solution (liq. $NH_3$)<br>E = -3184.4027221 | {$[Ga(NH_3)_6]I_2$}$^+$ solution (liq. $NH_3$)<br>E = -2860.0388569 |
| Ga -0.0149146114 0.044645939 -0.1386985085 | Ga -0.6394886628 -0.0065359819 -0.0101978265 |
| N -0.1687022064 0.1434269297 2.0095329418 | N 0.6079986049 1.384482969 0.9621338902 |
| N -2.0247254281 0.8263616522 -0.2294926974 | N 0.6351234958 0.1359585531 -1.6828236432 |
| N 0.2099206024 -0.068057362 -2.0395376219 | N -1.8868876198 -1.3964627756 -0.9837520176 |
| N 1.9518511323 -0.7097162068 0.2701899084 | N -1.913608691 -0.1458954193 1.6621446579 |
| N 0.8160287542 2.0215382872 0.0494872795 | N 0.6126880717 -1.5343551389 0.7191610524 |
| N -0.7991011465 -1.9435504149 -0.0988180432 | N -1.8922588336 1.5220567283 -0.7382459929 |
| H -0.9076396389 0.7513498628 2.3451780154 | H 0.4827004607 1.4251846956 1.9692201935 |
| H 0.7114232443 0.5189848267 2.3851646786 | H 0.5058090167 2.3370864935 0.6253149986 |
| H -0.3149590638 -0.7556644615 2.4548043965 | H 1.5953320261 1.1419038583 0.8138891458 |
| H 1.9817877219 -1.6476297301 0.653907447 | H -2.896270864 -0.1227584842 1.3625822362 |
| H 2.4807689669 -0.1111334707 0.9118795326 | H -1.8093619839 0.6139730003 2.3278043417 |
| H 2.4297418324 -0.7352983429 -0.6239992254 | H -1.8082290269 -1.0046887523 2.1937904182 |
| H 0.1321058273 2.7495020186 0.2242408616 | H 1.5993953067 -1.2706957531 0.6069992632 |
| H 1.4976911698 2.0612559885 0.8166364428 | H 0.4915709472 -1.737642186 1.7067812999 |
| H 1.3064147926 2.2807583911 -0.7996218277 | H 0.5075968912 -2.4195149965 0.2326246506 |
| H -1.6645803932 -1.9540353194 -0.6578944252 | H -1.7725104519 1.7259374973 -1.7259400701 |
| H -1.0118709175 -2.375505254 0.7928937746 | H -1.786591774 2.4067764768 -0.2509382461 |

| | |
|---|---|
| H -0.1486253847 -2.5581236454 -0.5770066241 | H -2.878634405 1.2582787273 -0.6249817461 |
| H -2.5469836224 0.9893829023 0.6241727185 | H 0.5348299168 0.9995376698 -2.2077195932 |
| H -2.0263686106 1.6986527832 -0.747005822 | H 1.6173109234 0.1048923236 -1.3836488704 |
| H -2.5693124 0.1592952997 -0.7939543608 | H 0.526834888 -0.6178761487 -2.3547317311 |
| H -0.590291412 -0.5102590226 -2.4804449068 | H -1.7614512095 -1.4348660478 -1.9908283709 |
| H 0.3128641212 0.8262833303 -2.5037285003 | H -2.8740743617 -1.1540560971 -0.834695216 |
| Cl -3.410930516 -1.5520716762 -1.9930846477 | H -1.7852664771 -2.3498951936 -0.6492021776 |
| Cl 2.7948001862 1.4649266953 2.610758214 | I 3.8912040101 -0.0079239579 0.032221166 |
| | I -5.1687461982 -0.0077350598 -0.0473808125 |

* lowest energy configuration of non-converged optimization run

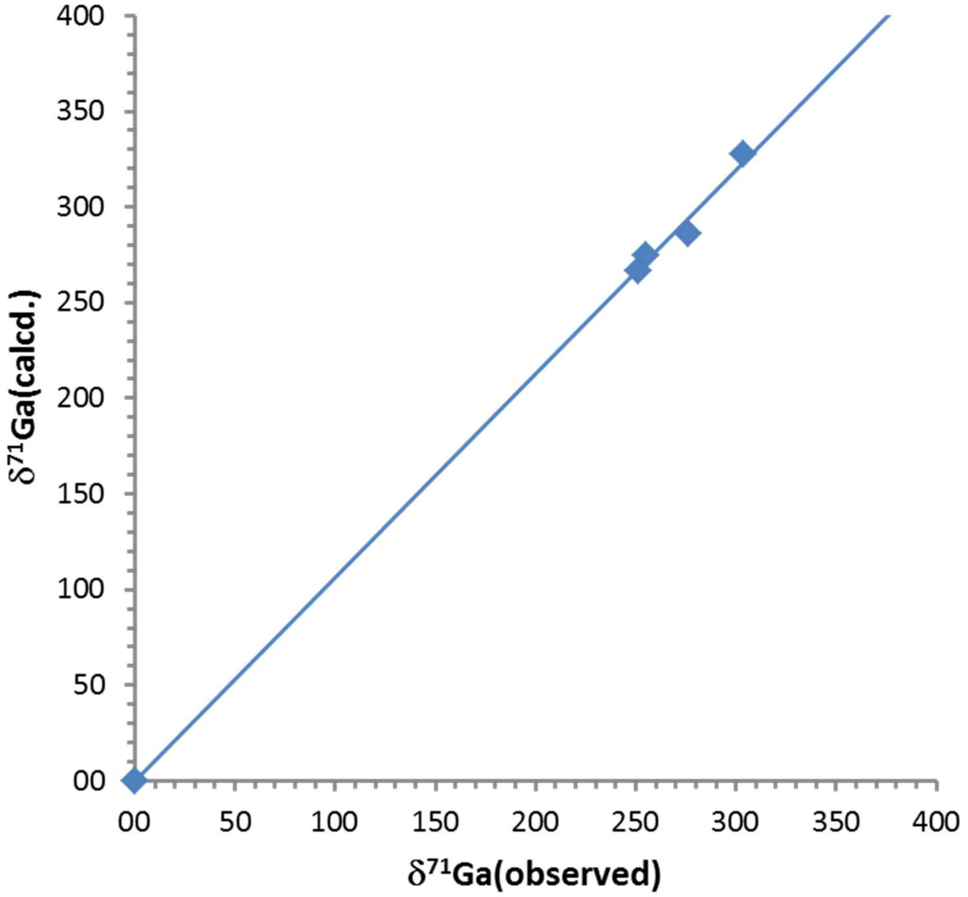


**Figure S3:** Plot of calculated vs. observed $^{71}$Ga NMR chemical shifts for selected reference compounds. Linear regression analysis gave a relation $\delta^{71}$Ga(calcd) = 1.0645· $\delta^{71}$Ga(obsd) - 0.1814 ($R^2$ = 0.9987, blue line).

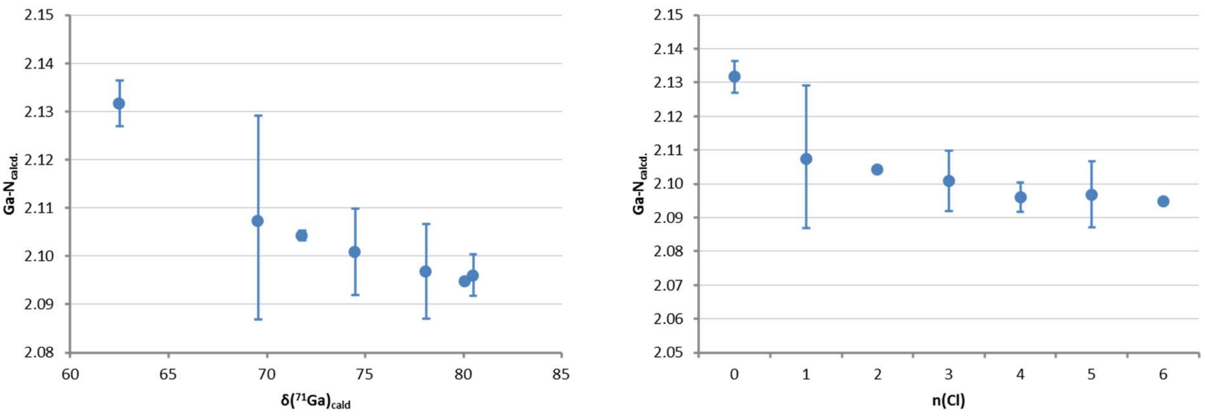


**Figure S4:** Plot of calculated average Ga–N distances vs. calculated $^{71}$Ga NMR chemical shifts (left) and the number *n* of chloride ligands (right) for aggregates $\{[Ga(NH_3)_6]Cl_n\}^{(3-n)+}$ ($n = 1 - 3$), $\{[Ga(NH_3)_6]Cl_n\}^{(n-3)-}$ ($n = 4 - 6$). Error bars denote standard deviation for all individual Ga–N distances in each single complex.

## 3. MD Simulations

All interactions between $NH_3$, $Ga^{3+}$, and $Cl^{-}$ in the molecular dynamics study were modeled by empirical interaction potentials. The corresponding potentials and force field parameters are denoted in Table S3 for non-bonded interactions, and Table S4 for intra-molecular interactions, respectively. For the Lennard-Jones parameters the Lorentz-Berthelot mixing rules were applied. While +3 and –1 were imposed for the $Ga^{3+}$ and $Cl^{-}$ ions, the atomic charges of N (–1.1091) and H (+0.3697) of the $NH_3$ molecules are adopted from a previous study.[S3]

**Table S3:** Non-bonded interaction parameters

**Lennard-Jones potential**

$$V = 4\varepsilon\left[\left(\frac{\sigma}{r}\right)^{12} - \left(\frac{\sigma}{r}\right)^{6}\right]$$

| i−j | ε (kJ/mol) | σ (Å) | reference |
|---|---|---|---|
| N−N | 0.03975 | 4.04468 | S3 |
| H−H | 0.04184 | 1.10650 | S3 |
| N−H | 0.04078 | 2.57559 | |
| Ga−H | 0.0 | 0.0 | |
| Cl−Cl | 2.52262 | 4.01261 | S4 |
| Cl−N | 0.31666 | 4.02864 | |
| Cl−H | 0.32488 | 2.55955 | |
| Ga−Cl | 9.12500 | 3.48630 | |

**Buckingham potential**

$$V = Ae^{-r/\rho} - \frac{C}{r^6}$$

| i−j | A (kJ/mol) | ρ (Å) | C(Å$^6$ kJ/mol) | reference |
|---|---|---|---|---|
| Ga−Ga | 585510.0 | 0.31846 | 24122.0 | S5 |
| Ga−N | 84178.0 | 0.31318 | 0.0 | S5 |

**Table S4:** Intra-molecular interaction parameters

**Bond stretching**

$$V = k(r - r_0)^2$$

| i−j | k (kJ/mol) | $r_0$ (Å) | reference |
|---|---|---|---|
| N−H | 4278.391 | 1.02 | S3 |

**Bond angle bending**

$$V = k(\theta - \theta_0)^2$$

| j−i−k | k (kJ/mol) | $\theta_0$ (°) | reference |
|---|---|---|---|
| H−N−H | 341.649 | 106.4 | S3 |

## 4. References


S1 Frisch, M. J.; Trucks, G. W.; Schlegel, H. B.; Scuseria, G. E.; Robb, M. A.; Cheeseman, J. R.; Scalmani, G.; Barone, V.; Petersson, G. A.; Nakatsuji, H.; Li, X.; Caricato, M.; Marenich, A.; Bloino, J.; Janesko, B. G.; Gomperts, R. Mennucci, B.; Hratchian, H. P.; Ortiz, J. V.; Izmaylov, A. F.; Sonnenberg, J. L.; Williams-Young, D.; Ding, F.; Lipparini, F.; Egidi, F.; Goings, J.; Peng, B.; Petrone, A.; Henderson, T.; Ranasinghe, D.; Zakrzewski, V. G.; Gao, J.; Rega, N.; Zheng, G.; Liang, W.; Hada, M.; Ehara, M.; Toyota, K.; Fukuda, R.; Hasegawa, J.; Ishida, M.; Nakajima, T.; Honda, Y.; Kitao, O.; Nakai, H.; Vreven, T.; Throssell, K.; Montgomery Jr., J. A.; Peralta, J. A.; Ogliaro, F.; Bearpark, M.; Heyd, J. J.; Brothers, E.; Kudin, K. N.; Staroverov, V. N.; Keith, T.; Kobayashi, R.; Normand, J.; Raghavachari, K.; Rendell, A.; Burant, J. C.; Iyengar, S. S.; Tomasi, J.; Cossi, M.; Millam, J. M.; Klene, M.; Adamo, C.; Cammi, R.; Ochterski, J. W.; Martin, R. L.; Morokuma, K.; Farkas, O.; Foresman, J. B.; Fox, J. D. Gaussian 09, Revison E.01; Gaussian Inc.: Wallingford **2009**.

S2 Hertrampf, J.; Schlücker, E.; Gudat, D.; Niewa, R. Dissolved Intermediates in Ammonothermal Crystal Growth: Stepwise Condensation of $[Ga(NH_3)_4]^-$ toward GaN. *Cryst. Growth Des.* **2017**, *17*, 4855–4863.

S3 D. Zahn, A molecular simulation study of the auto-protolysis of ammonia as a function of temperature, Chem. Phys. Lett. 682 (2017) 55–59.

S4 P. Li, L.F. Song, K.M. Merz, Systematic parameterization of monovalent ions employing the nonbonded model, J. Chem. Theory Comput. 11 (2015) 1645–1657.

S5 P. Zapol, R. Pandey, J.D. Gale, An interatomic potential study of the properties of gallium nitride, J. Phys. Condens. Matter. 9 (1997) 9517–9525.
